# Host heterogeneity and unpredictability in parasite outbreaks

**DOI:** 10.1073/pnas.2522557123

**Published:** 2026-01-15

**Authors:** Jacob A. Cohen, Mark Viney, Andy Fenton

**Affiliations:** ^a^Department of Evolution, Ecology and Behaviour, Institute of Infection, Veterinary and Ecological Sciences, University of Liverpool, Liverpool L69 7ZB, United Kingdom

**Keywords:** parasites, host heterogeneity, transmission

## Abstract

Experimental tests of the effects of host heterogeneities on parasite transmission are rare; previous studies focus on a single host trait over one generation of transmission. Thus, the long-term epidemiological consequences of interacting host heterogeneities remain unclear. We used a laboratory-based experimental system comprising the red flour beetle (*Tribolium castaneum*) infected with the eugregarine parasite, *Gregarina cloptoni*, to show, firstly, that two colonies of the beetle are heterogeneous in their susceptibility and infectiousness to the eugregarine. We then constructed experimental populations that differed in population-level heterogeneity in susceptibility and infectiousness by varying proportions of the colonies in the populations and tracked parasite transmission over eight weeks. We found that differences in parasite transmission among populations were explained by population mean susceptibility and infectiousness, rather than heterogeneity. Finally, to test the effects of heterogeneity on equilibrium parasite transmission we developed an agent-based model (ABM) for our host–parasite system. Results from our model showed that populations with the highest level of heterogeneity had the highest between-simulation variability in epidemiological measures. This demonstrated that even with the same starting conditions, host heterogeneities can drive high levels of uncertainty in epidemiological predictability. Our work presents a rare experimental assessment of how heterogeneities in susceptibility and infectiousness affect parasite transmission, showing that while these heterogeneities may not affect transmission directly, they can still affect epidemic variability, and hence predictability. This variability has considerable consequences for outbreak management strategies yet remains understudied, so further tests of the effects of host heterogeneity on epidemic variability will be important.

## Significance

Individuals can differ in how likely they are to acquire parasites (susceptibility) and to pass them on to others (infectiousness). Questions of how those differences affect the spread of parasites are often addressed using mathematical models, and there is little experimental work which has directly tested those predictions. Using a laboratory-based host–parasite system, we experimentally tested how parasite spread is affected by different levels of variation between individuals in susceptibility and infectiousness. We combined our experiments with modeling to show that variation among hosts did not affect parasite spread but did affect the predictability of measures of parasite spread. Future work must test how differences between hosts affect this predictability in more detail.

Host heterogeneities in parasite transmission are ubiquitous and can have a substantial impact on parasite transmission and epidemiology (1–5). Hosts can differ behaviorally, immunologically, genetically and physically (6–10), each of which can affect at least one of three key epidemiologically relevant host traits: susceptibility (the host’s propensity to become infected following parasite exposure), infectiousness (the capacity of an infected host to transmit parasites), and contact rate (the rate of transmission-relevant contacts). It is the combination of these traits that ultimately determines an individual host’s capacity to transmit parasites (11). Despite this conceptual understanding, it remains unclear how individual-level heterogeneities can scale up to affect population-level parasite transmission. Specifically, while there have been some theoretical explorations of the interactive effects of host heterogeneity in susceptibility and infectiousness on parasite transmission, empirical evidence of the interactive effects of heterogeneities in susceptibility and infectiousness on parasite transmission is lacking.

Previous modeling has shown that heterogeneity in host susceptibility can decrease peak and final epidemic sizes and reduce the force of infection relative to homogeneous host populations (12–14). Explicitly accounting for heterogeneity in susceptibility is often necessary for mathematical models to accurately replicate observed transmission rates (15–18). Conversely, models that incorporate heterogeneity in infectiousness have produced conflicting findings: some have shown that heterogeneity in infectiousness can reduce outbreak probability, frequency, size, and duration (12, 19, 20); others have found it reduces the efficacy of mitigation strategies, increases the basic reproduction number (*R_0_*), drives outbreaks, and increases variability in epidemic occurrence (21–23). More recently, it has been recognized that interactions among host heterogeneities (sometimes referred to as “coupled heterogeneities”) are important in driving parasite transmission (3, 12, 24, 25). For example, positive covariation between heterogeneities in susceptibility and infectiousness (within individuals, or at the population level) can shorten time to peak prevalence and increase *R_0_*, the likelihood of parasite establishment and final epidemic size (3, 12, 24–27). Conversely, negative covariation can reduce *R_0_*, epidemic size and probability of establishment, and slow the time to peak prevalence.

Despite this weight of theoretical evidence, these phenomena have not been fully investigated empirically. While some studies have experimentally manipulated host heterogeneity, they have generally done so for only a single trait and over a single generation of transmission and so do not test longer-term epidemiological consequences or the effects of interacting heterogeneities (28–30). Studies that track multiple-generations of transmission have used observational data, meaning that there is no experimental manipulation of populations (31). Therefore, it is still unknown what effects host heterogeneity in susceptibility and infectiousness have on parasite transmission, and whether theoretical predictions of these phenomena are correct. To achieve this level of understanding, studies are needed that include coupled heterogeneities, use a long-term experimental approach that directly manipulates levels of heterogeneity, and track the epidemiological consequences over multiple transmission cycles.

Here, we experimentally test the effect of host heterogeneity in susceptibility and infectiousness on long-term parasite transmission dynamics. We do this using a tractable, laboratory-based invertebrate host–parasite system where we manipulate population-level host heterogeneity in both susceptibility and infectiousness and track measures of transmission over multiple generations. The host in our system is the red flour beetle, *Tribolium castaneum* (Herbst 1797) (Coleoptera: Tenebrionidae), a common pest of stored cereal products globally. The parasite in our system is a eugregarine gut parasite, *Gregarina cloptoni* which, epidemiologically, shares much in common with macroparasites such as parasitic helminths (32); details in *SI Appendix*, section 1. Using this system we first quantify the heterogeneity in host susceptibility and infectiousness between two different laboratory colonies. We then combine those colonies in differing proportions to experimentally alter population-level heterogeneities in susceptibility and infectiousness and test the epidemiological consequences of those differing degrees of heterogeneity over multiple generations of parasite transmission. Finally, we use an agent-based model (ABM) to extend our experimental findings and determine how these coupled heterogeneities affect equilibrium parasite transmission.

## Results

### Two Colonies of *T. castaneum* are Heterogeneous in Their Susceptibility and Infectiousness to *G. cloptoni*.

We compared the susceptibility of two *T. castaneum* laboratory colonies to the eugregarine gut parasite *G. cloptoni* by exposing them to controlled doses of parasite-contaminated flour. We quantified susceptibility using two measures: parasite prevalence, the proportion of exposed larvae that become infected, and mean parasite intensity, the mean number of parasites per infected individual. The experiment was repeated three times.

**Fig. 1.**
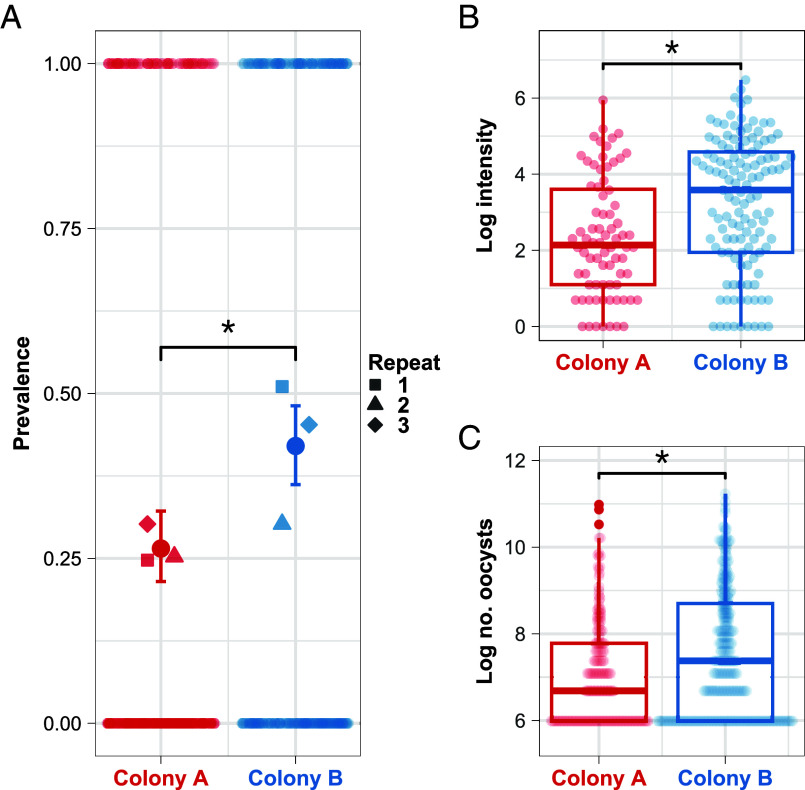
Colonies differ in host susceptibility (*A* and *B*) and infectiousness (*C*). (*A*) Infection prevalence per colony across the three experimental repeats. The circular points with error bars are the overall prevalence per colony across the three repeats and their respective binomial 95% CI. The lighter squares, triangles, and diamonds are the mean parasite prevalence per colony per repeat. (*B*) Logged individual infection intensities per colony across the three repeats. (*C*) Logged individual oocyst counts per colony across the six repeats. For all panels, the asterisks denote statistical significance (*P* < 0.05) and the lighter circular points show the raw data. For panels (*B* and *C*), the lower and upper limits of the boxes show the first and third quartiles of the data, respectively, and the median is shown by the horizontal line in the box. The upper whisker extends to the largest value that is no further than 1.5 times the interquartile range from the upper limit of the box, and points show all values beyond that.

Mean parasite prevalence for Colony A (26.5%) was significantly lower than Colony B (42.0%) (Fig. 1*A*, GLMM estimated difference Colony A *vs* Colony B = −0.699, *P* = 0.0001). There were no differences in prevalence between experimental repeats (post hoc GLMM estimate Repeat 1 *vs* Repeat 2 = 0.48061, *P* = 0.0798; Repeat 1 *vs* Repeat 3 = 0.00953, *P* = 0.9989; Repeat 2 *vs* Repeat 3 = −0.47107, *P* = 0.0876). Mean parasite intensity was also significantly lower for Colony A (mean intensity: 29.6 parasites per individual) than Colony B (mean intensity: 76.3) (Fig. 1*B*, post hoc GLMM estimated difference Colony A *vs* Colony B = −0.947, *P* < 0.0001). Intensity was marginally significantly higher for Repeat 1 compared to Repeat 2 (post hoc GLMM estimated difference Repeat 1 *vs* Repeat 2 = 0.592, *P* = 0.0474), but there were no other significant differences in intensity between repeats (post hoc Repeat 1 *vs* Repeat 3 = 0.148, *P* = 0.7869; Repeat 2 *vs* Repeat 3 = −0.444, *P* = 0.1675). In addition, within-colony variance in intensity was significantly higher in Colony B than Colony A (details in *SI Appendix*, section 6).

We next compared the infectiousness of the colonies by exposing larvae from each colony to controlled doses of parasite-contaminated flour, after which we held larvae individually to count the number of parasite infective stages (oocysts) present in their frass. This experiment was repeated six times. Oocyst shedding was significantly lower for Colony A (mean shedding: 1339.6 oocysts per individual) than Colony B (mean shedding: 2116.9 oocysts per individual) (Fig. 1*C*, post hoc GLMM estimated difference Colony A *vs* Colony B = −0.458, *P* = 0.0004). Oocyst counts for Repeat 1 were significantly different from Repeats 3 to 6 as were oocyst counts between Repeat 2 and Repeats 3 to 5 (*SI Appendix*, Table S1). Within-colony variance in oocyst shedding was significantly higher in Colony B than Colony A (details in *SI Appendix*, section 6).

In this host–parasite system, host susceptibility and infectiousness are linked due to the life cycle of the parasite, as shown by the positive correlation between individual-level intensity and oocyst counts (*SI Appendix*, Fig. S9, details in *SI Appendix*, section 5).

### Population-Level Mean Trait Values, Not Heterogeneity in Susceptibility and Infectiousness, Dominate Parasite Transmission.

**Fig. 2.**
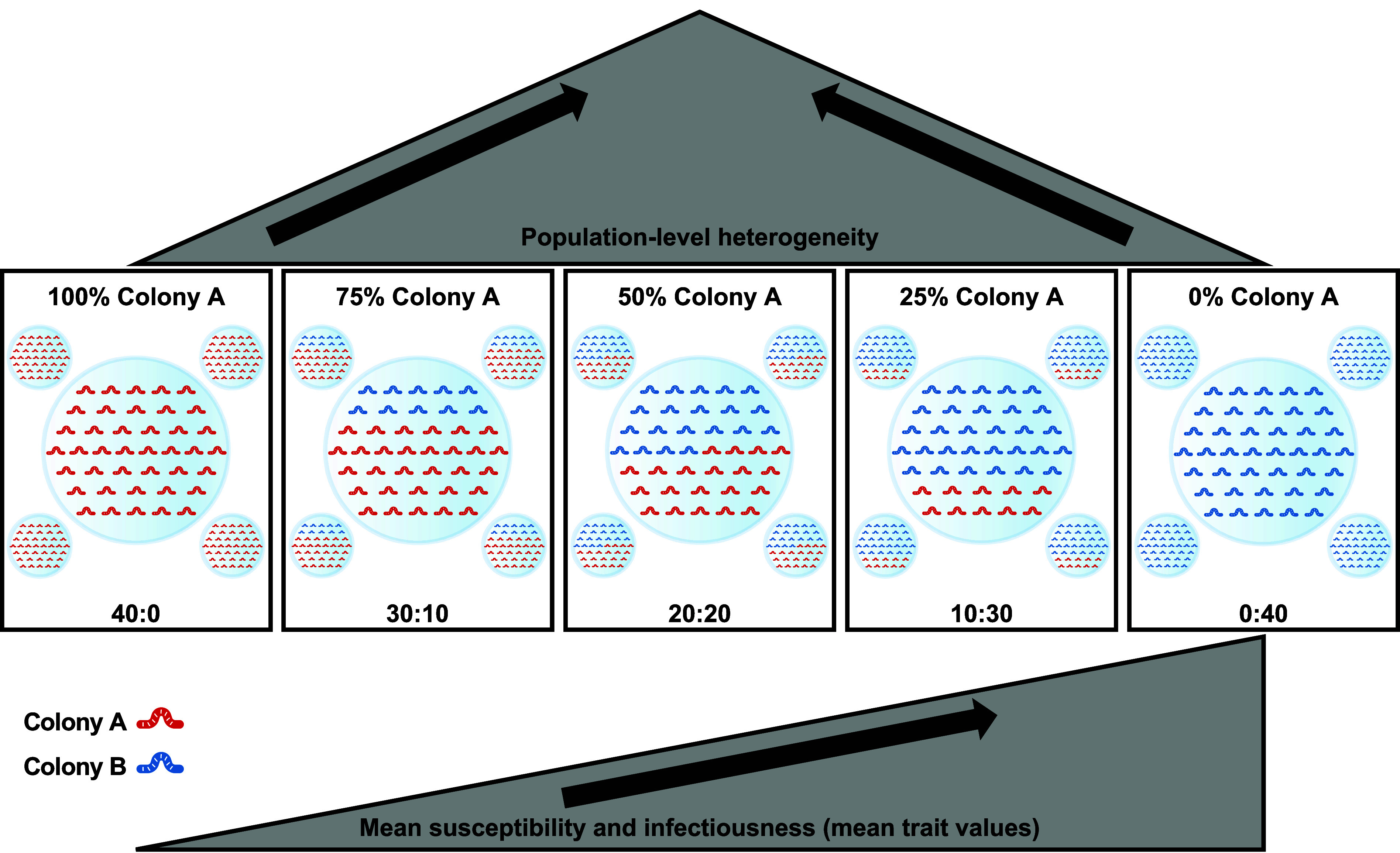
Schematic of experimental design for testing the effects of heterogeneity in susceptibility and infectiousness on parasite transmission. Each of the five experimental treatments are shown in their own box, with treatment name at the top and ratios of Colony A (red):Colony B (blue) at the bottom. Each treatment contained forty larvae and was replicated five times for a total of 25 experimental populations. Population-level heterogeneity was maximized at the 50% Colony A treatment, while mean susceptibility and infectiousness increased from 100% Colony A to 0% Colony A.

We assessed the long-term epidemiological consequences of host heterogeneities in susceptibility and infectiousness using five experimental treatments each of 40 larvae, where each treatment varied in the proportions of each colony, so that each treatment had different degrees of susceptibility and infectiousness. The treatments were 40:0 (Colony A:Colony B, hereafter 100% Colony A), 30:10 (75% Colony A), 20:20 (50% Colony A), 10:30 (25% Colony A), and 0:40 (0% Colony A). Each treatment was replicated five times, for a total of 25 experimental populations. We considered the 100 and 0% Colony A treatments to be homogeneous, while the other three were heterogeneous. Population-level heterogeneity was maximized in the 50% Colony A treatments and declined toward the two homogeneous extremes. Conversely, the mean susceptibility and infectiousness in a treatment (henceforth “mean trait values”) increased as the proportion of Colony A larvae decreased (i.e., from 100 to 0% Colony A, Fig. 2). It should be noted that we could not quantify the heterogeneity for the mixed-colony treatments directly, because it is impossible to ascertain the specific trait combinations present among individuals allocated to each of these mixed-colony populations.

We exposed each experimental population to parasite-contaminated flour at the beginning of the experiment. We then tracked parasite transmission in each population over eight weeks, spanning multiple generations of transmission, and measured parasite prevalence and mean intensity through the experiment. To disentangle the effects of heterogeneity and mean trait values on parasite transmission, we compared our observed data with null expectations of prevalence and intensity of the heterogeneous treatments, based on proportional interpolation from the two homogenous values (100 and 0% Colony A; see Methods for details). Our null expectation was that parasite transmission would increase linearly with mean trait values. However, if population-level heterogeneity affects transmission, we would expect a departure from this linear relationship. We considered there to be evidence for a deviation from the null expectation if the experimental results for a heterogeneous treatment fell outside of the 95% CI of the calculated null expected value.

**Fig. 3.**
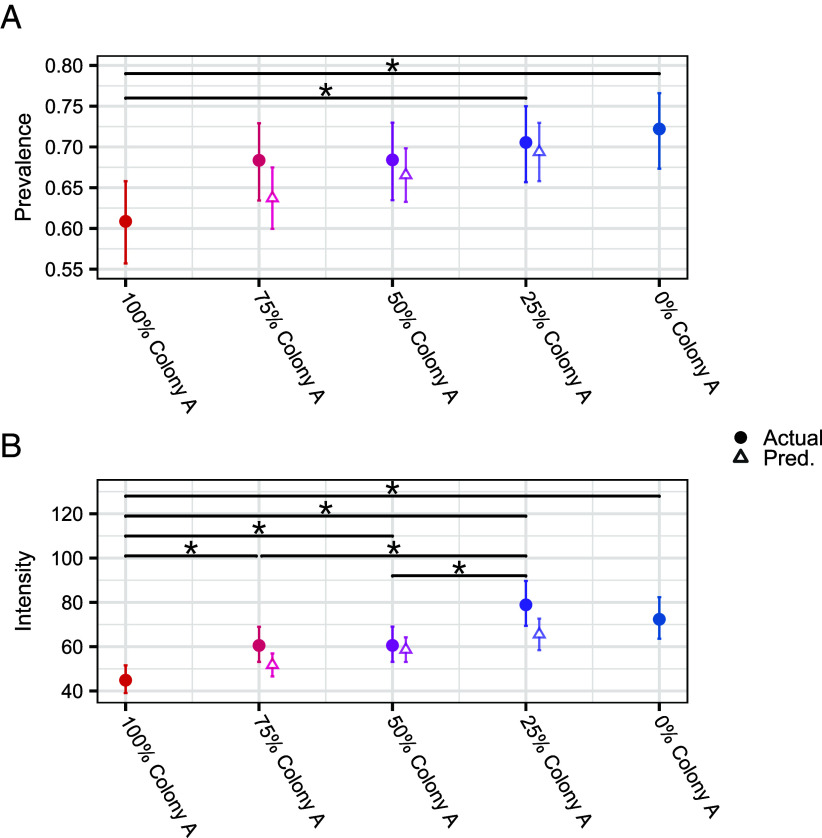
Experimental test of the effects of host heterogeneity in susceptibility and infectiousness on parasite transmission. GLMM estimates for (*A*) overall mean prevalence and (*B*) overall mean intensity across the 8 wk experiment are denoted with circular points. Asterisks denote statistically significant differences between treatments. Triangular points show the null expected values for the three heterogeneous treatments. All error bars are 95% CI. We dissected 10 larvae per experimental population per week. There were five experimental populations per treatment, thus 50 larval dissections per treatment per week, or 400 per treatment over the eight weeks of the experiment.

There was a general trend for infection prevalence to increase with mean trait values (Fig. 3*A* and *SI Appendix*, Fig. S1). Prevalence in the 100% Colony A treatment was significantly lower than in the 25 and 0% Colony A treatments (Fig. 3*A* and *SI Appendix*, Table S2). There was no evidence of a deviation from the null expectation that parasite transmission increased linearly with mean trait values. Specifically, 95% CIs for the model estimates of the three heterogeneous treatments all overlapped with their corresponding expected values, though actual values were always higher than null expected values (Fig. 3*A*).

As with prevalence, there was a general trend for infection intensity to increase with mean trait values (Fig. 3*B* and *SI Appendix*, Fig. S2). There were significant differences in intensity between six pairwise treatments (Fig. 3*B* and *SI Appendix*, Table S3). We also found no evidence of deviation from the null expectation that parasite transmission increased linearly with mean trait values; the 95% CIs for the intensity measures of the three heterogeneous treatments all overlapped with their corresponding null expected values, though actual values were again always higher than null expected values (Fig. 3*B*).

### Variability in Equilibrium Transmission Increases With Heterogeneity.

We used a stochastic, discrete-time ABM to extend our experimental results to further test the effects of host heterogeneity in susceptibility and infectiousness on equilibrium measures of parasite transmission. Our ABM modelled individual *T. castaneum* larvae (the “agents”) belonging to the two colonies, in 1d timesteps, with the environmentally transmitted parasites. The model was parametrized with our experimentally determined estimates of colony mean prevalence (Fig. 1*A*), which determined the probability of parasite establishment in individuals of each colony.

We replicated the transmission experiment (Fig. 2) as closely as possible. However, our model allowed us to increase sample sizes (120 total agents) in each simulation of the five experimental treatments (initial proportions of Colony A:Colony B) to levels that were experimentally intractable. Each of the five experimental treatments was simulated 250 times, thus giving 1,250 simulations in total. We ran each simulation for 1,500 timesteps and considered simulations to have reached equilibrium when we observed that an essentially static value had been achieved for 100 timesteps. All simulations had reached equilibrium by 500 timesteps. At each timestep, we tracked population-level parasite prevalence, mean parasite intensity, and mean agent age (inversely related to parasite-induced mortality; host mean age is low when the accumulation of lethal levels of infection is faster).

**Fig. 4.**
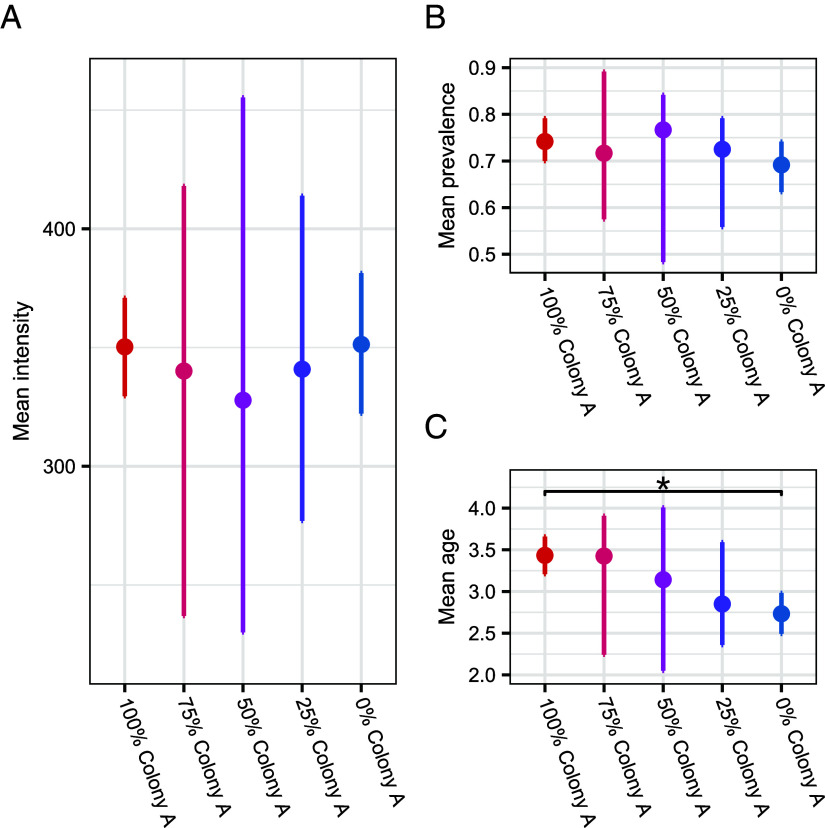
ABM simulation results. Points show median (50th centile) equilibrium (timesteps 500 to 1,500) values of 250 simulations for each treatment of population mean (*A*) intensity, (*B*) prevalence, and (*C*) agent age, for the different experimental scenarios. Error bars are 90% CIs (5th to 95th centiles) of the predicted mean equilibrium values (timesteps 500 to 1,500) across the 250 simulations. Asterisks denote significant differences between experimental scenarios.

Predicted equilibrium mean intensities and mean prevalences did not differ among the treatments (overlap in 90% CIs; Fig. 4 *A* and *B*). The only measure that did differ between treatments was mean agent age, which was higher in the 100% Colony A treatment than the 0% Colony A treatment (Fig. 4*C*), due to high levels of parasite-induced mortality in the 0% Colony A treatment. Despite no detectable effect of population-level heterogeneity on mean infection levels in our model, there was a clear effect of heterogeneity on the between-simulation variance in equilibrium measures of infection (Fig. 4 *A*–*C*). Specifically, between-simulation variance was consistently smallest for the homogeneous treatments (100 and 0% Colony A) and increased with increasing heterogeneity, so that the 50% Colony A treatment (with the maximum amount of heterogeneity) had the largest variance. Pairwise Levene’s tests for the homogeneity of variance confirmed the significant differences in between-simulation variance for all pairwise comparisons of the three measures (all pairwise *P*-values < 0.0001 after correcting for multiple comparisons). Thus, increasing population-level host heterogeneity in susceptibility and infectiousness resulted in higher variability in the predicted outcomes of parasite infection.

## Discussion

How, or indeed, if host heterogeneity affects parasite transmission is an unresolved question in disease ecology. Existing theory has suggested that it can, though this has not been experimentally validated. Using a tractable experimental host–parasite system, extended with agent-based modeling, we did not find clear evidence that host heterogeneity affected widely used measures of parasite infection, but we did find that host heterogeneity can drive epidemic variability and hence the predictability of epidemiological outcomes.

Our experimental work showed that mean trait values appear sufficient to explain much of the observed transmission pattern in our system. However, several features of our system may also dampen detectable effects of coupled heterogeneities, including parasite-induced developmental delays, differences in within-colony variance and individual-level positive correlation between susceptibility and infectiousness (details in *SI Appendix*, sections 4, 6, and 5, respectively). For instance, the positive correlation between susceptibility and infectiousness would suggest that any effects of heterogeneity in the mixed-colony treatments could arise from both between- and within-colony variation. In addition, the difference in within-colony variance for both susceptibility and infectiousness meant that we could not fully separate the independent effects of mean trait values and heterogeneity on transmission. Though, our null-expectation analysis (linear interpolation between homogeneous extremes) would have detected nonlinear departures if heterogeneity (which shows a unimodal relationship with treatment) played an important role in driving transmission. As the heterogeneous treatments did not deviate from the null expectations, and a power analysis showed that our experimental design was highly sensitive to detecting among-treatment differences in prevalence and intensity (details in *SI Appendix*, section 7), we conclude that mean trait values largely explain the average levels of prevalence and intensity we observed. However, we cannot rule out that some of the observed effects were influenced by differences in variance rather than mean alone, particularly since measured values of prevalence and intensity were consistently higher (though non-significantly) than their corresponding null expected values. Future studies could address this limitation by constructing experimental populations that independently manipulate mean and variance, for example, by sampling individuals from multiple colonies with overlapping trait distributions, or by assembling populations that share similar mean trait values but differ in variance.

Parasite transmission is dependent on host susceptibility, infectiousness, and contact rate (11). We explicitly manipulated susceptibility and infectiousness in our experiments, but assumed homogeneity of behavior between and within the colonies. Larval movement and behavior of *T. castaneum* remain understudied, so we cannot rule out the possibility that behavioral variation between colonies contributed to differences in exposure and transmission. Future work quantifying movement and behavior would help clarify the relative contributions of behavioral versus physiological sources of heterogeneity between the colonies.

Importantly, although our ABM did not include between-colony differences in variance, it nonetheless demonstrated that increasingly heterogeneous host populations led to greater uncertainty in predicted epidemiological outcomes. These findings are supported by previous work showing that host heterogeneity in infectiousness can lead to more variability in the chances of an epidemic occurring (23) and increased variability in measures of transmission, including size of peak infection and the effective reproduction number (33). Our work demonstrates that even with the same population-level starting conditions, inherent individual-level host heterogeneities can drive great uncertainty in parasite transmission outcomes. These finding have clear real-world relevance in predicting epidemic events in human and animal populations, because forecasting and predicting epidemics is essential for the effective implementation of control policies (34).

Epidemic predictability has been studied in specific disease systems such as measles, chickenpox, and pertussis (35) and is often assumed to be driven by parameters such as population size or stochastic fluctuations in epidemiological or environmental processes (36, 37). We suggest that the drivers of unpredictability in parasite transmission are understudied and that more testing will need to be undertaken in this area to understand how host heterogeneities can interact to drive unpredictability in parasite transmission.

Our empirical work did not show evidence for increased between-replicate variability as heterogeneity in the treatments increased (details in *SI Appendix*, section 9), whereas predictions from our ABM did. This difference may be due to the low power of our empirical work to detect among-treatment variance (details in *SI Appendix*, section 7), or it may reflect the different temporal scales and dynamical states captured by each approach. Our empirical experiment ran over eight weeks, encompassing several generations of transmission but was unlikely to have reached equilibrium conditions. In contrast, our analysis of the ABM explicitly focused on long-term, equilibrium dynamics, though analysis of the initial dynamics of our ABM showed similar trends to our empirical experiment (*SI Appendix*, Fig. S8; details in *SI Appendix*, section 3). Our modeling results therefore suggest that the influence of host heterogeneity on epidemiological variability may become most apparent at or near equilibrium, or at least over longer time scales, whereas shorter-term empirical studies may capture primarily the mean-driven dynamics of early epidemic phases. Together, these findings indicate that heterogeneity may have limited effects on transmission in the short term but can influence long-term predictability once equilibrium dynamics are reached. This distinction has practical implications: short-term outbreak data may underestimate the influence of host heterogeneity on epidemic predictability, highlighting the importance of considering longer-term or equilibrium dynamics when modeling disease spread and evaluating control strategies.

Pending further study, we can only speculate what might be driving epidemic unpredictability in our system, though we suggest that it may be driven by higher variance in the force of infection (FOI) early in the epidemics for the most heterogeneous populations. Individual-level contributions to the FOI (measured as the number of environmental parasite stages produced per individual) were most variable when heterogeneity in susceptibility and infectiousness was maximized (50% Colony A treatment). It is well established that FOI is often key to understanding the epidemiological dynamics of host–parasite systems (38, 39), so higher variability in the FOI in the first few timesteps could drive larger differences in equilibrium parasite transmission.

Under our design, coupled heterogeneities may have been insufficient to alter patterns of parasite transmission, even though they likely influence higher-order dynamics such as variability and predictability. Previous work has shown that host heterogeneities may drive parasite transmission differently, dependent on the way in which host infectiousness is determined (25). The parasite we used can be considered a macroparasite (32), which most closely matches the “donor dependent” process of infectiousness determination of (25). Under this scenario, we would expect transmission to be dominated by maximum levels of host infectiousness, rather than coupled heterogeneities between susceptibility and infectiousness (25); a prediction largely upheld by our results. Alternative mechanisms of infectiousness determination can generate other relationships between heterogeneity and transmission (25), hence it would be intriguing to test those predictions with other host–parasite systems (*e.g.,* calves infected with bovine viral diarrhoea virus (40) or snails infected with the flatworm *Schistosoma mansoni* (30)).

In summary, our work presents a rare experimental assessment of how population-level host heterogeneities in susceptibility and infectiousness affect multiple generations of parasite transmission. We show that such heterogeneities may not affect parasite transmission and rather that this is driven predominantly by population-level mean trait values. But we do find that population-level heterogeneities can affect epidemic variability, and hence predictability. This epidemic variability has considerable consequences for outbreak management strategies, and it will be important to test the effects of host heterogeneity on epidemic variability in more detail.

## Materials and Methods

### Experimental System.

All experiments were conducted using the larval stage of two distinct laboratory colonies of *T. castaneum* and a eugregarine gut parasite, *G. cloptoni*. We used larvae because previous experiments have shown that eugregarine burden and subsequent shedding were higher in larvae than adults (41). Further details of the experimental system are in the *SI Appendix*.

### Assessing Host Heterogeneity in Susceptibility.

On experimental Day 0, 120 larvae of the same colony were placed in a 90-mm diameter petri dish containing parasite-contaminated flour. The negative control was 120 larvae with parasite-free flour. Larvae were left on the flour for 24 h, removed using a 300 µm mesh sieve (ASTM no. 50) and placed on 10 g parasite-free flour, where they stayed for the remainder of the experiment.

Beginning on Day 4 and ending on Day 7, 24 experimental and 12 control larvae from each colony were removed each day and held individually in wells of 96-well plates for 48 h after which they were dissected and the number of parasites in each larva counted (details in *SI Appendix*, section 2.3). Daily dissection order was randomized. Larvae were omitted from all analyses if they had pupated, died, or were badly dissected; numbers of omitted larvae were Repeat 1: n = 4 (1.39%), Repeat 2: n = 3 (1.06%) and Repeat 3: n = 1 (0.34%) of all attempted dissections. This experiment was repeated on three separate occasions. No control larvae were found to be infected throughout any experimental repeat. *SI Appendix*, Fig. S3 shows the experimental design, and *SI Appendix*, Table S4 provides further experimental details.

To determine the effect of colony and experimental repeat on parasite intensity and parasite prevalence, we used two generalized linear mixed models (GLMMs). One model had PARASITE INTENSITY as the response variable (negative binomial distribution), while the other model used PARASITE PRESENCE/ABSENCE as the response variable (binomial distribution). The explanatory terms were COLONY and EXPERIMENTAL REPEAT (fixed effects) and DAY SAMPLED (random effect). Colony estimates and pairwise comparisons were done using post hoc estimate marginal means (EMMs), which corrects for multiple comparisons.

### Assessing Host Heterogeneity in Infectiousness.

This experiment was repeated six times, with details of differences between experimental repeats provided in *SI Appendix*, Table S5. Larvae were infected as for assessing host heterogeneity in susceptibility (above). After allowing parasites to establish and develop, larvae were held individually in wells of 96-well plates, and then wells were examined for the presence of gametocysts 24 h later using a stereo microscope. If gametocysts were present in the well, the larva was removed and the number of gametocysts was counted using the stereo microscope.

Gametocysts were then left for 24 h to dehisce and release oocysts. To determine the number of oocycts, 40 µL of PBS was added to the dehisced gametocysts, mixed vigorously for 10 s to evenly distribute the oocysts in the solution, and then 10 µL of the solution was placed into a hemocytometer slide, and the number of oocysts counted (details in *SI Appendix*, section 2.4). The order in which gametocyst and oocyst counts were done was randomized each day. Larvae that had died, pupated, or did not produce oocysts were omitted from all analyses. *SI Appendix*, Fig. S4 shows the experimental design.

We defined individual infectiousness as the total number of oocysts produced by an individual larva. We used a GLMM (Poisson distribution) to determine whether infectiousness differed between colonies, which incorporated an observation level random effect (OLRE) to account for overdispersion in the data (42) and where OOCYST COUNT was the response variable, and explanatory variables were COLONY, EXPERIMENTAL REPEAT (fixed effects), DAY SAMPLED, and OLRE (random effects). We generated colony estimates and conducted pairwise comparisons using post hoc EMMs.

### Testing the Effects of Host Heterogeneity on Parasite Transmission.

We set up populations of 40 larvae which varied in the proportion from each colony (henceforth called treatments): 40:0 (100% Colony A), 30:10 (75% Colony A), 20:20 (50% Colony A), 10:30 (25% Colony A), and 0:40 (0% Colony A). Each treatment was replicated five times, for a total of 25 experimental populations.

On experimental Day-1, 10 larvae from each colony were dissected to confirm that the naive larvae being used for the experiment were not infected. Experimental populations were then prepared by generating the treatments with larvae from each colony in a 60 mm diameter petri dish containing 5 g of parasite-free flour sieved through a 250 µm mesh sieve. On Day 1, larvae were transferred to 5 g of parasite-contaminated flour, left for 24 h and then removed to 5 g of parasite-free flour. Ten larvae from each petri dish (n = 250 larvae total) were then removed and placed in individual wells of a 96-well plate for 24 h. These 250 larvae were subsequently dissected for parasite counts on Day 2 (details in *SI Appendix*, section 2.5).

This experiment ran over an eight-week period, which is longer than the *T. castaneum* larval stage [17 to 20 d at 29 °C (43)], so petri dishes were refreshed with new, naive larvae as the older larvae pupated. This was done by obtaining larvae as previously (details in *SI Appendix*, section 2.1) and adding them to the experimental population. Following the initial dissection on Day 2, there was a consistent weekly schedule for the remainder of the experiment that is described in detail in the *SI Appendix*.

We used two generalized linear mixed models (GLMMs) to analyze the data: the first had PARASITE PRESENCE/ABSENCE as the response variable (binomial distribution); the second model had PARASITE INTENSITY as the response variable (negative binomial distribution). Explanatory variables for both models were DISSECTION WEEK and TREATMENT (fixed effects), and EXPERIMENTAL POPULATION (random effect). We generated mean overall estimates (across the eight weeks) for the prevalence and intensity of each treatment using EMMs.

We tested whether heterogeneity was driving infection levels in the heterogeneous treatments by generating null expectations based on infection levels from the homogenous treatments. We did this using the mean overall estimates for the two homogeneous treatments to generate random distributions with the same number of samples as the experimental data; details are in the *SI Appendix*.

We considered the results from an experimental treatment to be a deviation from the null expectation if there was no overlap in the 95% CI of the GLMM model estimates and the 95% CI of the null expectation.

### ABM.

The model was a stochastic, discrete-time model comprised of “agents” representing individual beetle larvae run in discrete 1-d timesteps. The model tracked the colony identity of the agent, and at each time step the number of i) parasite infective stages an agent was exposed to ($P_{upt.}$), ii) fully established parasites per agent ($P_{est.}$), iii) parasite infective stages in the environment ($P_{env.}$), and iv) the age of each agent (in timesteps). Parameters for the model are detailed in *SI Appendix*, Table S6.

Agents were assigned a probability of pupating in every timestep based on their age, with the probability assumed to follow a sigmoid relationship (*SI Appendix*, Eq. **S2**). Upon pupation, agents were removed and replaced with an agent of the same colony with age 0. The threshold for parasite-induced host mortality was 1,000 parasites, after which agents were removed and replaced with an agent of the same colony with age 0.

The number of parasite infective stages each agent was exposed to at each timestep ($P_{upt.}$) was randomly selected from a Poisson distribution with a mean equal to $P_{exp.}\times P_{env.}$, where $P_{exp.}$ was the parasite exposure rate per agent, per timestep. The number of parasite infective stages taken up by each agent was then subtracted from $P_{env.}$. $P_{env.}$ was also reduced by a constant per timestep parasite mortality rate, $P_{mort.}$.

Parasites taken up by an agent in the previous timestep were given a probability of establishment in the agent in the current timestep, which was determined using random binomial number generation, with the number of trials equal to the number of parasites taken up in the previous timestep and the probability of success equal to that agent’s susceptibility value (a colony-dependent measure, σ; values in *SI Appendix*, Table S6). The resulting number of successes was used as the number of parasites that successfully established in that agent for that timestep ($P_{est.}$), out of those it had been exposed to in the previous timestep. Parasites that failed to establish were lost and had no opportunity to establish in future timesteps.

We assumed that all parasites that successfully established also successfully replicated. Successful parasites released parasite-infective stages two timesteps after establishing. The number of parasite infective stages released by an infected host was calculated by multiplying the number of successful parasites infecting that host by a randomly generated Poisson distributed number with mean ι (the expected infectiousness of the larvae, details in *SI Appendix*, Table S6). Thus, highly infected hosts would release high numbers of infective stages and so, across the host population, susceptibility and infectiousness will be positively correlated, where a colony with a higher susceptibility would also have a higher infectiousness.

We replicated our experimental setup described in the previous section as closely as possible but increased sample sizes to 120 total agents. Further details of the model are presented in the *SI Appendix*.

## Supplementary Material

Appendix 01 (PDF)

## Data Availability

Code, experimental data have been deposited in Github (https://dx.doi.org/10.5281/zenodo.17697172) (44).
