# Supplementary material for: Host heterogeneity and unpredictability in parasite outbreaks
Source: Proc Natl Acad Sci U S A. 2026 Jan 15;123(3):e2522557123. doi: 10.1073/pnas.2522557123 (PMC12818443; doi:10.1073/pnas.2522557123)
Supplement: Supplementary file 1 — Appendix 01 (PDF) [file pnas.2522557123.sapp.pdf]

Supplementary Information

1. Background – *Tribolium castaneum* and eugregarines

The life cycle of eugregarines is described in detail by Logan, Janovy Jr and Bunker (1) and Clopton (2), and is shown in Figure S5. Briefly, the cycle begins with the parasite infectious particle, called an oocyst. This oocyst contains approximately eight sporozoites and is deposited in the food substrate (usually flour) of the beetle from previously infected hosts. Naïve hosts (either larvae or adults) then become infected by consuming oocyst-contaminated flour. The sporozoites then activate in the host gut and attach to the host epithelial gut cells, grow and become trophonts (also called trophozoites). The trophont continues to grow before detaching from the host epithelial cells to form a reproductive association with another trophont, thus becoming a gamont. When this gamont is mature it undergoes syzygy becoming a gametocyst by secreting a gametocyst wall. This gametocyst is then shed into the environment in the host's faeces. The gamonts inside the gametocyst wall undergo schizogony to generate gametes, which then fuse to form zygotes. The zygotes then secrete oocyst walls, undergo zygotic meiosis and form haploid sporozoites. Once the formation of the sporozoites within their oocyst is complete, the gametocyst bursts through a process known as dehiscence, and the oocysts are released back into the environment. One gametocyst will tend to produce thousands of oocysts.

Key aspects of the eugregarine life cycle that relate to susceptibility occur within the host. Specifically, whether (i) sporozoites are able to establish and become trophonts, and (ii) trophonts are able to reproduce with another parasite and produce a gametocyst. Host infectiousness is related to the number of oocysts (the parasite infective stage) that are released from parasites of that host. It has been shown that *T. castaneum* that have been previously exposed to eugregarines are less susceptible than eugregarine-naïve beetles, and that there is a genetic basis to beetle immune response to eugregarines (3, 4). However, little else is known about the susceptibility and infectiousness of *T. castaneum* infected with eugregarines.

2. Supplementary Methods

2.1. Experimental System

The two *T. castaneum* colonies used in these experiments, as well as four additional colonies not used in these experiments, were established in Liverpool in November 2022 and are derived from colonies that were collected, and previously maintained, by the Tate Lab at Vanderbilt University (4). The eugregarine parasite was originally isolated from infected *T. castaneum* beetles collected from a feed store in Kentucky, USA in June 2017 that we identified as *Gregarina cloptoni*, described below.

Stock colonies, as well as beetles used in all experiments, were maintained in full darkness at 30°C and 60% relative humidity. Beetles were housed in, and fed on, a medium of 95% sterilised organic whole wheat flour and 5% brewer's yeast. Colonies were kept in 1 litre glass jars covered with filter paper to allow for gas exchange in cultures containing eggs, larvae, pupae and adults.

We produced a new generation of each colony every six to eight weeks to prevent overlapping generations of beetles. We did this by placing adult beetles on flour media sieved through a 250 μm mesh sieve (ASTM no. 60) to lay eggs. After 48 hours, the beetles were removed from the flour media using a 710 μm mesh sieve (ASTM no. 25). This allowed the eggs to hatch and develop into adults of the next generation. Colony generations were tracked and recorded. We counted generation as the number of generations since the colonies were first established in Liverpool.

To generate larvae for experiments, beetle eggs were collected by placing adult *T. castaneum* in a 90 mm diameter petri dish for 48 hours with flour sieved through a 250 μm mesh sieve (ASTM no. 60). Adults were then removed and the flour containing the eggs was left in the petri dish for a further 8-10 days to allow the eggs to hatch into larvae to use for the experiment.

The parasites were maintained in a continuously infected colony of *T. castaneum* established in Liverpool November 2022 that derived from an equal mix of the six colonies initially

established in Liverpool. Infections were initiated using parasite-contaminated flour generated from gametocysts (and ultimately oocysts) released by infected larvae and adult beetles maintained on flour. Before use, parasite-contaminated flour was sieved through a 300 µm mesh sieve (ASTM no. 50) to remove all beetles and then homogenised in a spice grinder to produce an even distribution of oocysts throughout the flour. Parasite-contaminated flour was prepared no more than four days before use in an experiment.

2.2. Parasite identification

To identify the eugregarine parasite, gametocysts were collected from an infected *T. castaneum* larva by holding it individually in the well of a 96-well plate for 24 hours, allowing it to produce frass containing gametocysts. Gametocysts were collected by putting 10 µL of phosphate-buffered saline (PBS) into the well, mixing it for 10 seconds, and then removing the PBS and placing it in an Eppendorf tube.

DNA was extracted from the pooled gametocyst sample after Criado-Fornelio*, et al.* (5). Briefly, 190 µL of distilled water was added to a 10 µL gametocyst sample and held at 100°C for two minutes. We used this template DNA to amplify a fragment of the small subunit (SSU) rDNA using universal eukaryotic PCR primers from F1 5'-GCGCTACCTGGTTGATCCTGCC-3' and R1 5'-GATCCTTCTGCAGGTTCACCTAC-3' with the following conditions: 4 cycles of initial denaturation at 94°C for 4:30 min, 45°C for 1 min and 72°C for 1:45 min; 34 cycles of 94°C for 0:30 min (denaturation), 50°C for 1:00 min (annealing), 72°C for 1:45 min (extension); final extension period at 72°C for 10:00 min (6). The PCR product was then isolated using the Bioline Isolate II Plasmid Mini Kit (Meridian Bioscience, Cincinnati, Ohio, USA) and sequenced by Eurofins Genomics (Ebersberg, Germany).

NCBI BLAST of the resulting sequence matched to a partial sequence of the SSU rRNA coding gene of *Gregarina cloptoni* (E-value: 0.0, GenBank Accession number FJ459742). *G. cloptoni* is a eugregarine species originally identified and described from parasites found in the guts of

beetles from a colony of *Tribolium freemani* at the University of Nebraska Lincoln in Lincoln, Nebraska, USA (7). Phylogenetic analysis shows that *G. cloptoni* is most closely related to *G. niphandrodes* and *G. polymorpha*, both of which are known to infect *Tenebrio molitor* (mealworm beetle) (8).

2.3. Assessing host heterogeneity in susceptibility

Larval dissections were undertaken by holding larvae individually, without food, in wells of 96-well plates for 48 hours to allow them to clear their guts of all food and parasites that had not yet established in the host (4). After this period, the number of parasites in each larva was counted by dissecting it in 10 µL of PBS to reveal the guts, which were then stained with 15 µL of Lugol's iodine and examined under a compound microscope with a 100x magnification (4). Parasite counts were separated by size and developmental stage into trophonts, sexually fused gamonts, and gametocysts (Figure S6) (9).

2.4. Assessing host heterogeneity in infectiousness

Oocysts counts were undertaken by counting the number of oocysts present on the middle square of the haemocytometer slide. All oocysts touching the middle line of the top and left triple border lines were included in the count. Oocysts touching the triple line on the bottom and right triple border lines were excluded from the count. The middle square of the haemocytometer is 1 mm^2 and there is 0.1 mm clearance between the cover slip and slide, so the volume of the solution in the middle square is 0.1 µL. The number of oocysts in the full 40 µL sample was therefore calculated by multiplying the number of oocysts in the middle square by 400. Figure S7 shows an oocyst chain.

2.5. Testing the effects of host heterogeneity on parasite transmission

Larval dissections were done as above, but larvae were starved for 24 hours, rather than 48 hours. The consistent weekly schedule for the remainder of the experiment following the initial dissection on Day 2 was as follows:

Day 1: 10 naïve larvae from each colony were dissected to ensure they were parasite free. The number of larvae still present in each experimental petri dish was counted, and the necessary number of new, naïve larvae added to the petri dish to maintain the correct proportion of colonies colony for that treatment. If the number of larvae needing to be added at the correct proportions led to non-integers, the colony with the larger proportion in the treatment was rounded up to the whole number. This was alternated weekly for the rest of the experiment. When the number of larvae needing to be replaced was an odd number for the 50% Colony A treatment, the final larva was not added, keeping the number of larvae added from each colony even.

Day 2: 10 larvae from each experimental petri dish were randomly selected by selecting the two larvae closest to the centre point and the 12, 3, 6, 9 o'clock positions around a 250 μm sieve. These larvae were placed in individual wells of a 96-well plate to be starved in advance of being dissected. This was done for all 25 experimental petri dishes in a randomised order.

Day 3: Larvae that had been starved for 24 hours were dissected and the number of parasites in their guts counted, for a total of 250 dissections.

Day 4: 10 naïve larvae from each colony were dissected to ensure they were parasite free. The number of larvae still present in each group was then counted, pupated larvae removed, and the necessary number of new, naïve, larvae calculated and added to the group. On occasions where the number of larvae needing to be added was not an integer, a coin flip determined which colony was rounded up.

Days 5, 6, 7: Experimental petri dishes left alone.

Null expectations for the heterogeneous treatments were calculated using the mean overall estimates for the two homogeneous treatments to generate random distributions with the same number of samples as the experimental data; binomial for prevalence and negative binomial for intensity (using the dispersion parameter from the relevant GLMM). We then calculated the mean value from each set of random numbers ($\bar{A}$, 100% Colony A; $\bar{E}$, 0% Colony A), before the predicted values for the 75% Colony A ($B_{exp.}$), 50% Colony A ($C_{exp.}$) and 25% Colony A ($D_{exp.}$) were calculated as

$$B_{exp.} = \bar{A} + (0.25 \times (\bar{E} - \bar{A})),$$

$$C_{exp.} = \bar{A} + (0.50 \times (\bar{E} - \bar{A})),$$

$$D_{exp.} = \bar{A} + \left(0.75 \times (\bar{E} - \bar{A})\right). \qquad (S1)$$

We ran this process 10,000 times for both prevalence and intensity before calculating the mean and 95% CI. We considered there to be a deviation from the null expectation for a treatment if there was no overlap in the 95% CI of the GLMM model estimates and the 95% CI of the null expectation. We used this analysis, rather than a GLM, because null expectations were generated from the means of 10,000 randomly generated distributions, meaning that it is not statistically rigorous to compare the actual, individual-level experimental data with means from the randomly generated distributions.

3. Agent-based model (ABM) additional information

The equation for the probability of pupation was

$$p(x) = \frac{1}{1 + e^{-(x - t_{pup.})}}, \qquad (S2)$$

where x was host age and $t_{pup.}$ was the age at which the pupation rate was 50% (10).

All parameters from Table S6 were kept constant in each of the simulations, apart from $No.\,Colony\ A$. We calculated equilibrium averages per treatment for all metrics by calculating the 50th centile for all simulations for timesteps 500-1,500. 90% CIs were generated by calculating the 5th and 95th centiles.

To aid comparison with the empirical results, we calculated non-equilibrium, early-stage means (timesteps 0-60) for each treatment for all metrics, using the same methods used for the equilibrium dynamics. Results showed a similar trend for predicted intensity by the ABM as for the empirical experiment; specifically, intensity increased as the proportion of Colony A decreased (Figure S8A). Prevalence was approximately equal for all treatments (Figure S8B). There were no statistically significant differences between any treatments.

4. Assessing the cost of parasitism

We assessed whether exposure to the parasite altered beetle development rates, and whether this effect differed between the beetle colonies. To analyse whether treatment (exposed *vs.* unexposed to the parasite) affected the proportion of pupated larvae per day, we used the binomial outcome of whether each individual larva had pupated or not on every day of Repeats 1 and 2 from the infectiousness experiment. These data were analysed using a binomial GLM with explanatory variables of EXPERIMENT DAY, COLONY, EXPERIMENTAL REPEAT and two interaction terms, EXPERIMENT DAY*TREATMENT and COLONY*TREATMENT.

Larval development rates differed significantly between exposed and unexposed (GLM Control vs. Experiment estimate = 0.963, $p < 0.0001$), even when accounting for differences between colonies. Unexposed larvae developed significantly faster than exposed larvae (Figure S9). Both interaction terms were significant (EXPERIMENT DAY*TREATMENT, p-value: 2.29e-08; COLONY*TREATMENT, p-value: 8.22e-05).

5. Assessing covariation between host susceptibility and infectiousness

To determine whether there was a relationship between individual-level susceptibility and infectiousness, we quantified the relationship between oocyst count (a measure of the infectiousness of an individual) and gametocyst count (a measure of the susceptibility of an individual) using data from Repeats 1 and 2 from the infectiousness experiment. We used a GLMM with a gamma distribution and log link function with a response variable of oocyst count and explanatory variables of NUMBER OF GAMETOCYSTS, COLONY, EXPERIMENTAL REPEAT and an interaction term NUMBER OF GAMETOCYSTS*COLONY (fixed effects) and DAY SAMPLED (random effect). Gametocyst count showed a significant positive relationship with oocyst count (GLMM estimate: 0.0163, p-value: 0.0231) (Figure S10). The interaction term was not significant, meaning that this relationship did not vary between the two colonies. This finding demonstrates that there was positive covariation between host susceptibility and infectiousness in our host-parasite system.

6. Comparing within-colony variance

To test whether within-colony variance in susceptibility (intensity) differed between Colonies A and B we used Levene's test for homogeneity of variance (median-centred), which confirmed that Colony A had a significantly lower variance than Colony B ($p = 0.002$, Figure S11, Table S7).

To test whether within-colony variance in infectiousness (oocyst shedding) differed between Colonies A and B we used Levene's test for homogeneity of variance (median-centred), which confirmed that Colony A had a marginally significantly lower variance than Colony B ($p = 0.046$, Figure S12, Table S8).

7. Power Analysis

To evaluate the statistical power of our long-term experiment, we conducted simulation-based power analyses for both prevalence (binomial GLMM) and intensity (negative-binomial GLMM). Power was estimated as the proportion of 2,000 simulations in which the treatment effect was significant (α = 0.05) based on a likelihood ratio test.

For prevalence, we simulated individual infection outcomes as Bernoulli draws with colony-specific probabilities estimated from the susceptibility experiment (Colony A: 0.26; Colony B: 0.42). Each simulated dataset comprised all treatments and replicates, and we fitted a binomial GLMM with prevalence as the response variable and explanatory variables of TREATMENT (fixed effect) and EXPERIMENTAL POPULATION (random effect). Our experimental design and sample sizes showed an 80.1% probability of detecting differences in prevalence among treatments.

For intensity, we simulated parasite counts as draws from a negative-binomial distribution parameterised with estimates from the susceptibility experiment (dispersion parameter: 0.605 (Colony A); 0.562 (Colony B), mean intensities: 29.6 (Colony A); 76.3 (Colony B)). We first tested for the probability of detecting differences in mean intensity among treatments using a negative binomial GLMM with intensity as the response variable and explanatory variables of TREATMENT (fixed effect) and EXPERIMENTAL POPULATION (random effect). This model assumed a constant variance across treatments. Our experimental design and sample sizes showed an 95.8% probability of detecting differences in mean intensity among treatments.

We then tested the probability of detecting differences in the variance in intensity among treatments fitting a second negative binomial GLMM with intensity as the response variable and explanatory variables of TREATMENT (fixed effect) and EXPERIMENTAL POPULATION (random effect). However, this model allowed the dispersion parameter of the negative binomial distribution to vary by treatment. We compared the constant variance and variable variance models using a likelihood ratio test. We considered there to be evidence for differences in the

variance in intensity among treatments when the model fit was significantly better for the variable variance model than the constant variance model. Our experimental design and sample sizes showed an 54.4% probability of detecting differences in the variance in intensity among treatments.

Overall, the power analyses indicate that our experimental design and sample sizes were sensitive to among-treatment differences in prevalence and mean intensity, but less sensitive to differences in the variance in intensity.

8. Testing for treatment-by-time differences in the empirical experiment

We tested whether the effects of host heterogeneity on parasite transmission varied across experimental weeks by comparing two GLMMs for each response variable (prevalence and intensity). For both prevalence (binomial distribution) and intensity (negative binomial distribution) the explanatory variables in the first GLMM were DISSECTION WEEK and TREATMENT (fixed effects), and EXPERIMENTAL POPULATION (random effect). The second GLMM also included a DISSECTION WEEK*TREATMENT (fixed effect), while all other explanatory variables were the same as the first GLMM.

We compared models using the Akaike Information Criterion (AIC) and likelihood ratio tests. AICs were lower for the models without the interaction term (prevalence AIC: 2335.3 *vs* 2343.8; intensity AIC: 13456 *vs* 13467). Likelihood ratio tests also showed significant differences between the two models (prevalence: $p = 0.0123$; intensity: $p = 0.0226$). Therefore, the simpler models without interaction terms were better supported, indicating no evidence for a treatment-by-time interaction in either prevalence or intensity.

9. Testing for differences in between-replicate variance of treatments

To determine whether there was empirical support for the patterns observed in our ABM we tested whether between-replicate variance differed among treatments in our empirical data.

To test for heterogeneity in between-replicate variance in prevalence between experimental treatments we calculated weekly prevalence for each of the 25 experimental populations. We then compared treatment-level variance per week (PREVALENCE ~ TREATMENT + DISSECTION WEEK), as well as over the entire length of the experiment (PREVALENCE ~ TREATMENT) using Levene's test for homogeneity of variance (median-centred). There was no evidence for heterogeneity in between-replicate variance in prevalence for either weekly ($p = 0.977$) or overall data ($p = 0.2009$).

To test for heterogeneity in between-replicate variance in intensity we calculated weekly mean intensity for each of the 25 experimental populations. We then compared treatment-level variance per week (MEAN INTENSITY ~ TREATMENT + DISSECTION WEEK), as well as over the entire length of the experiment (MEAN INTENSITY ~ TREATMENT) using Levene's test for homogeneity of variance (median-centred). There was no evidence for heterogeneity in between-replicate variance in intensity for either weekly ($p = 0.1344$) or overall data ($p = 0.1282$).

10. Software packages

Unless otherwise noted, all work was undertaken in R (11). GLMMs were run using the lme4, MASS and glmmTMB packages (12-14). Model diagnostics were carried out using the DHARMa package (15). Post hoc testing and model estimated mean values were run using the package emmeans (16). Other packages used in R were reshape (17), car (18) and tidyverse (19). The ABM was developed in NetLogo and simulations were run using NetLogo's Behaviorspace software tool (20). Plots were generated in R using packages ggplot2 (21), showtext (22), ggpubr (23), ggbeeswarm (24), ggsignif (25) and patchwork (26).

Supplementary Figure 1

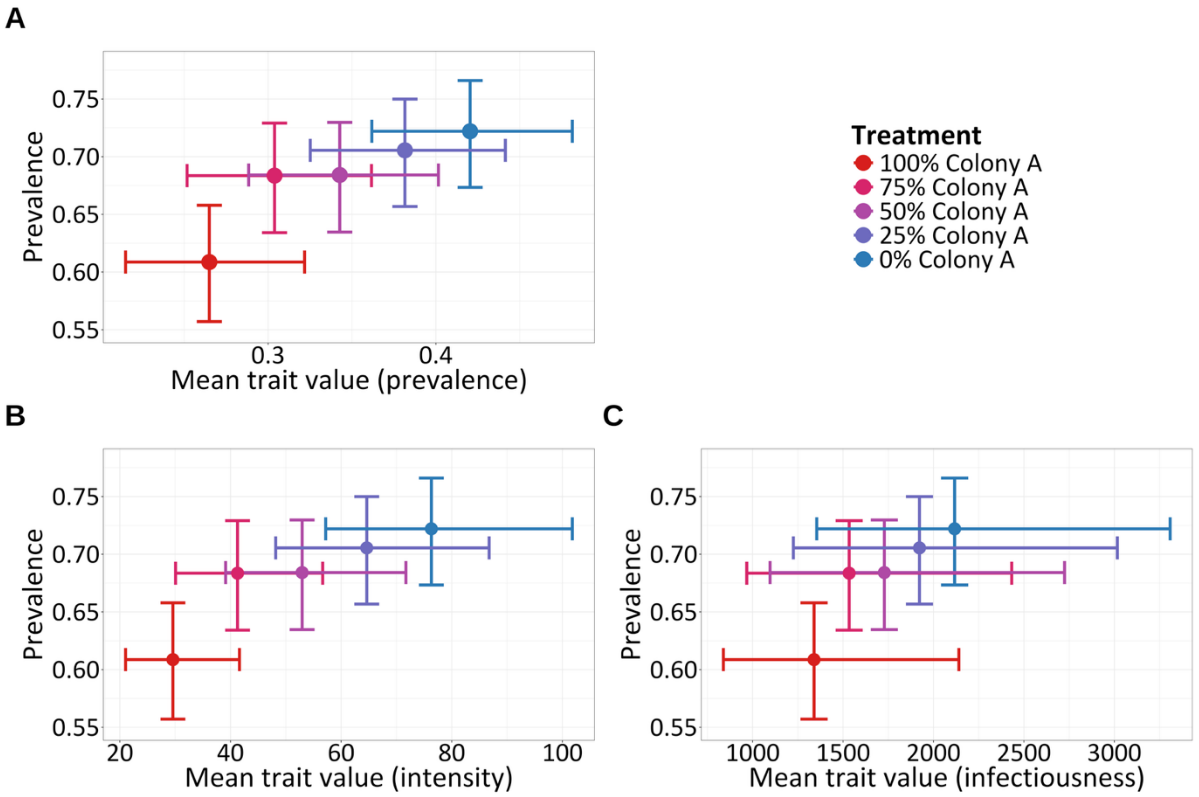

Figure S1. Effect of mean trait value on prevalence. (A) Mean prevalence value per treatment, (B) mean intensity per treatment, (C) mean infectiousness (oocyst count) per treatment. Colours denote treatment in all panels. Error bars show 95% CIs. There was a general trend where prevalence increased with mean trait value. Mean trait values (with 95% CIs) were calculated as

$$A = Colony\ A\ GLMM\ estimate$$

$$E = Colony\ B\ GLMM\ estimate$$

$$B = A + (0.25 \times (E - A)),$$

$$C = A + (0.50 \times (E - A)),$$

$$D = A + \big(0.75 \times (E - A)\big),$$

where A was the GLMM output for Colony A from the initial assays for susceptibility (prevalence and intensity) and infectiousness, B was the 75% Colony A treatment, C was the 50% Colony A treatment, D was the 25% Colony A treatment and E was GLMM output for Colony B from the initial assays for susceptibility (prevalence and intensity) and infectiousness.

Supplementary Figure 2

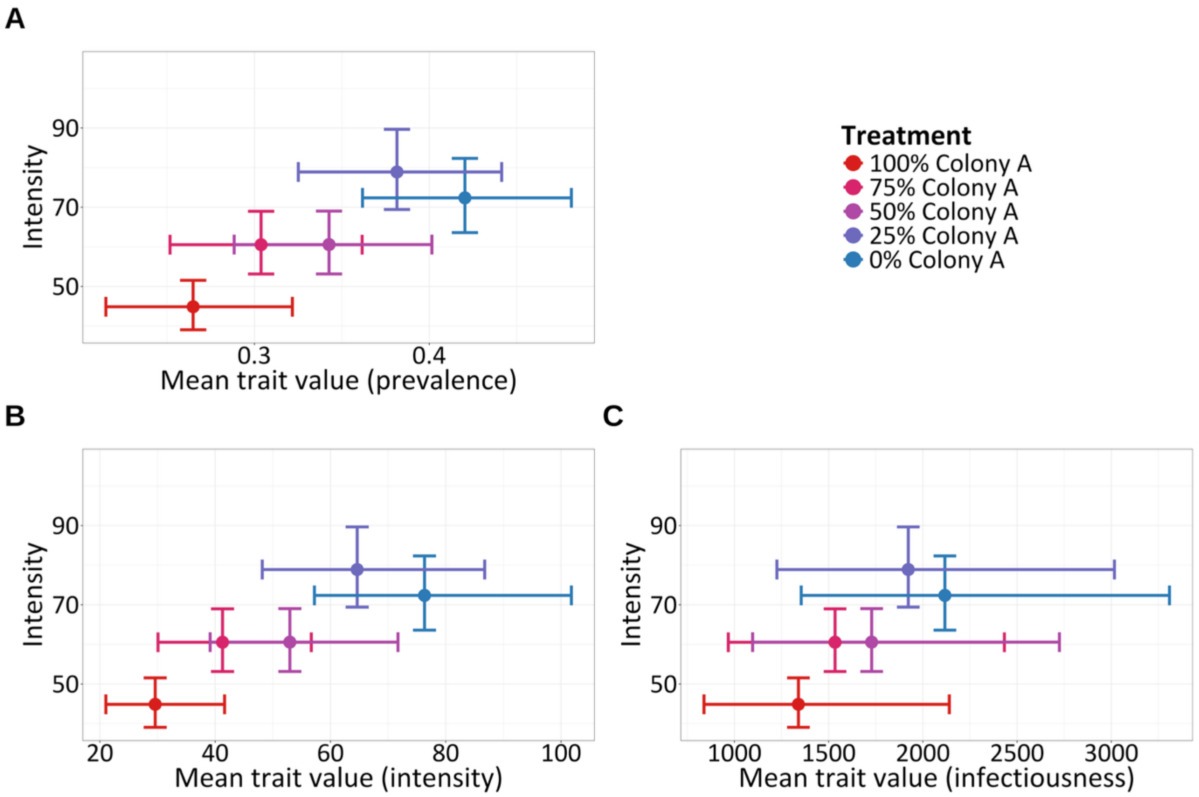

Figure S2. Effect of mean trait value on intensity. (A) Mean prevalence value per treatment, (B) mean intensity per treatment, (C) mean infectiousness (oocyst count) per treatment. Colours denote treatment in all panels. Error bars show 95% CIs. There was a general trend where intensity increased with mean trait value. Mean trait values were calculated as per Figure S1.

Supplementary Figure 3

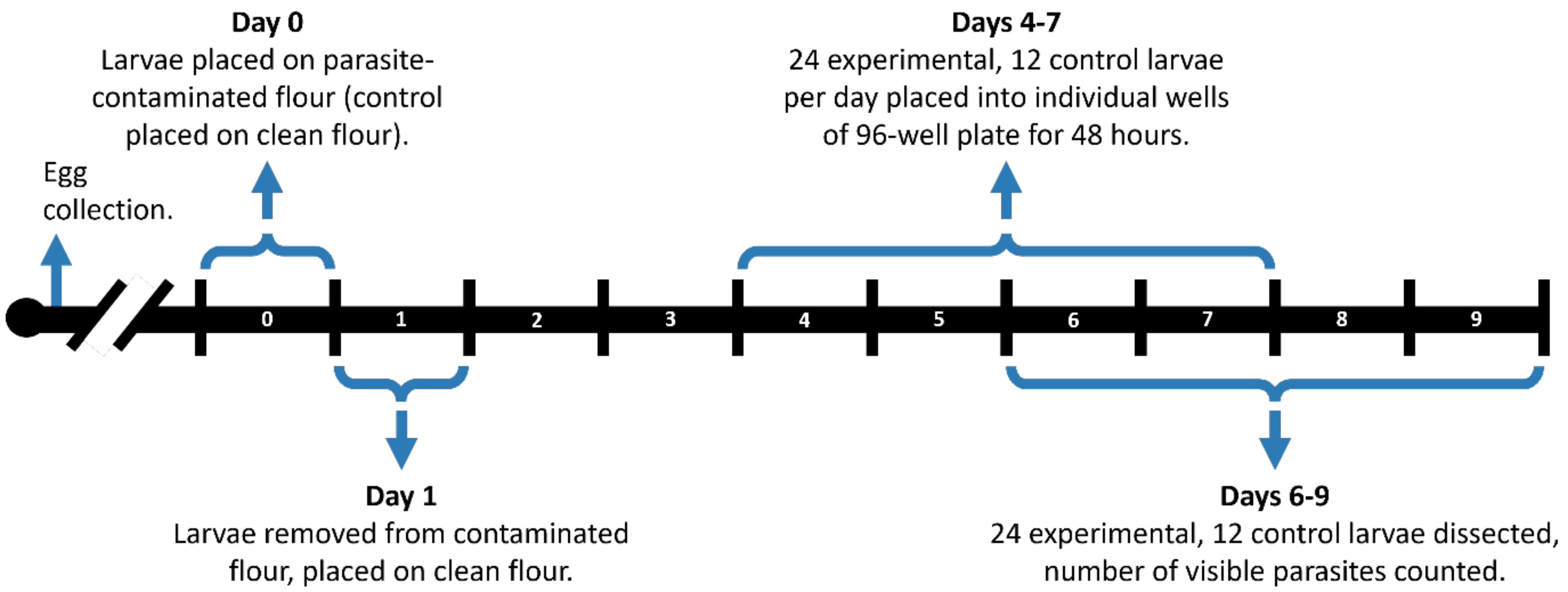

Figure S3. Schematic of experimental design for assessing host heterogeneity in susceptibility.

Supplementary Figure 4

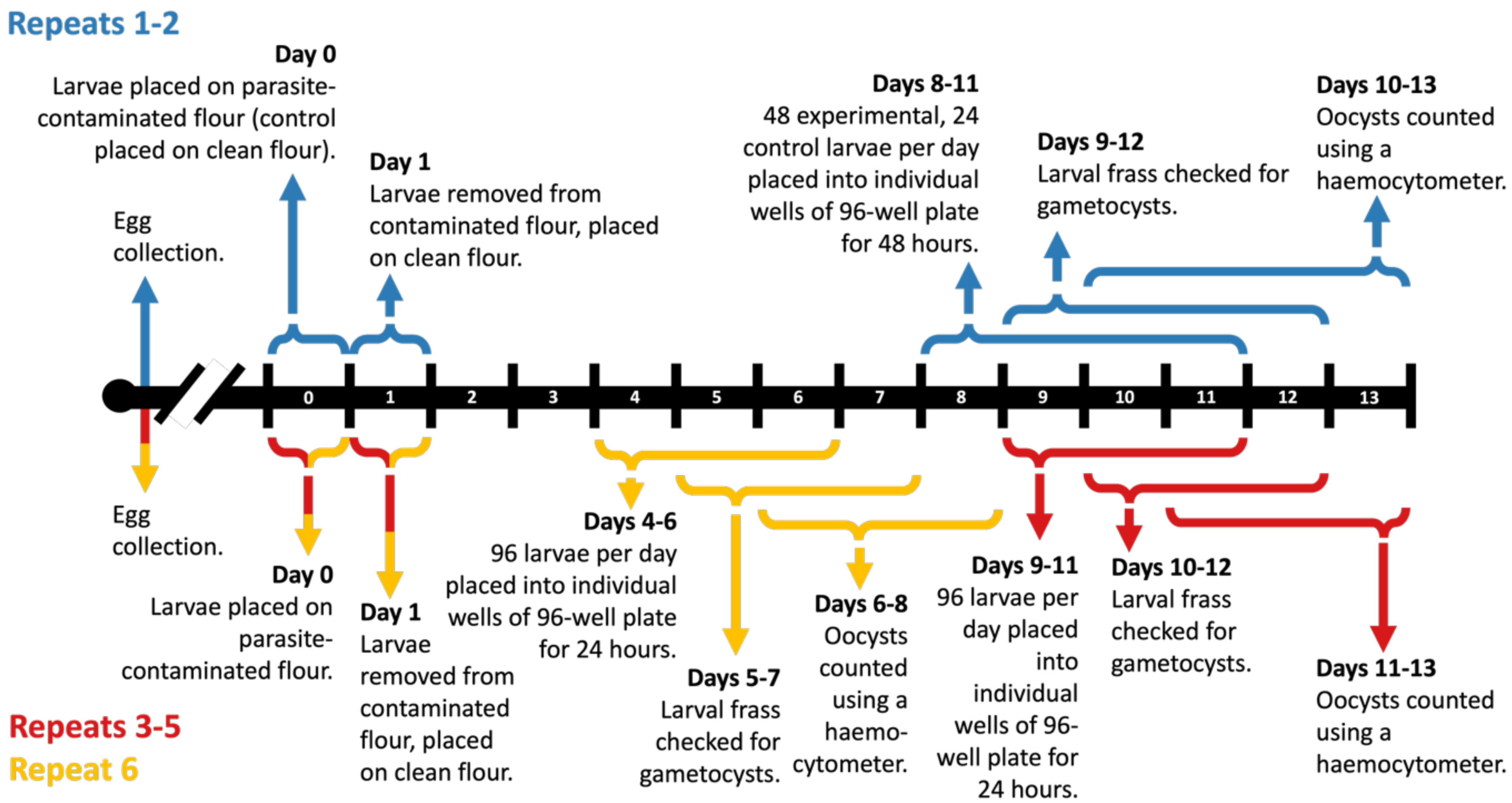

Figure S4. Schematic of experimental design for assessing host heterogeneity in infectiousness.

Supplementary Figure 5

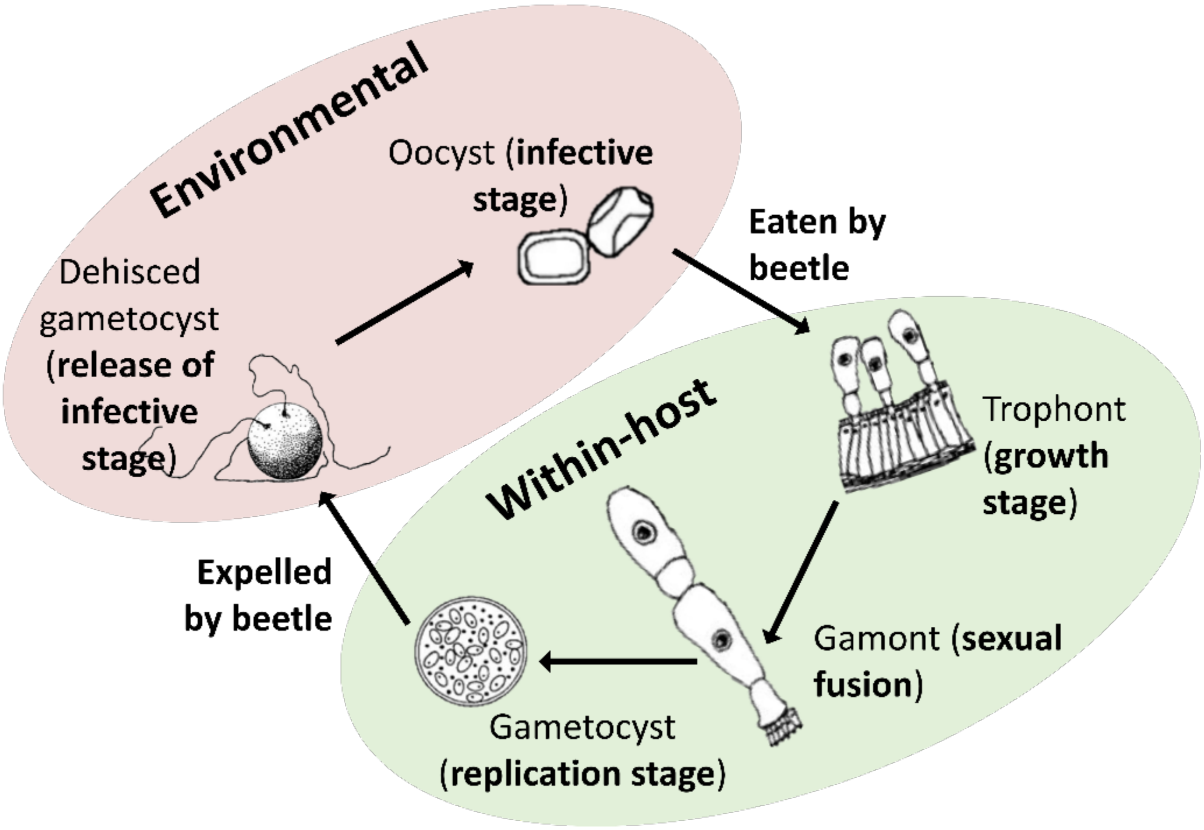

Figure S5. Eugregarine life cycle, drawings from Clopton (2).

Supplementary Figure 6

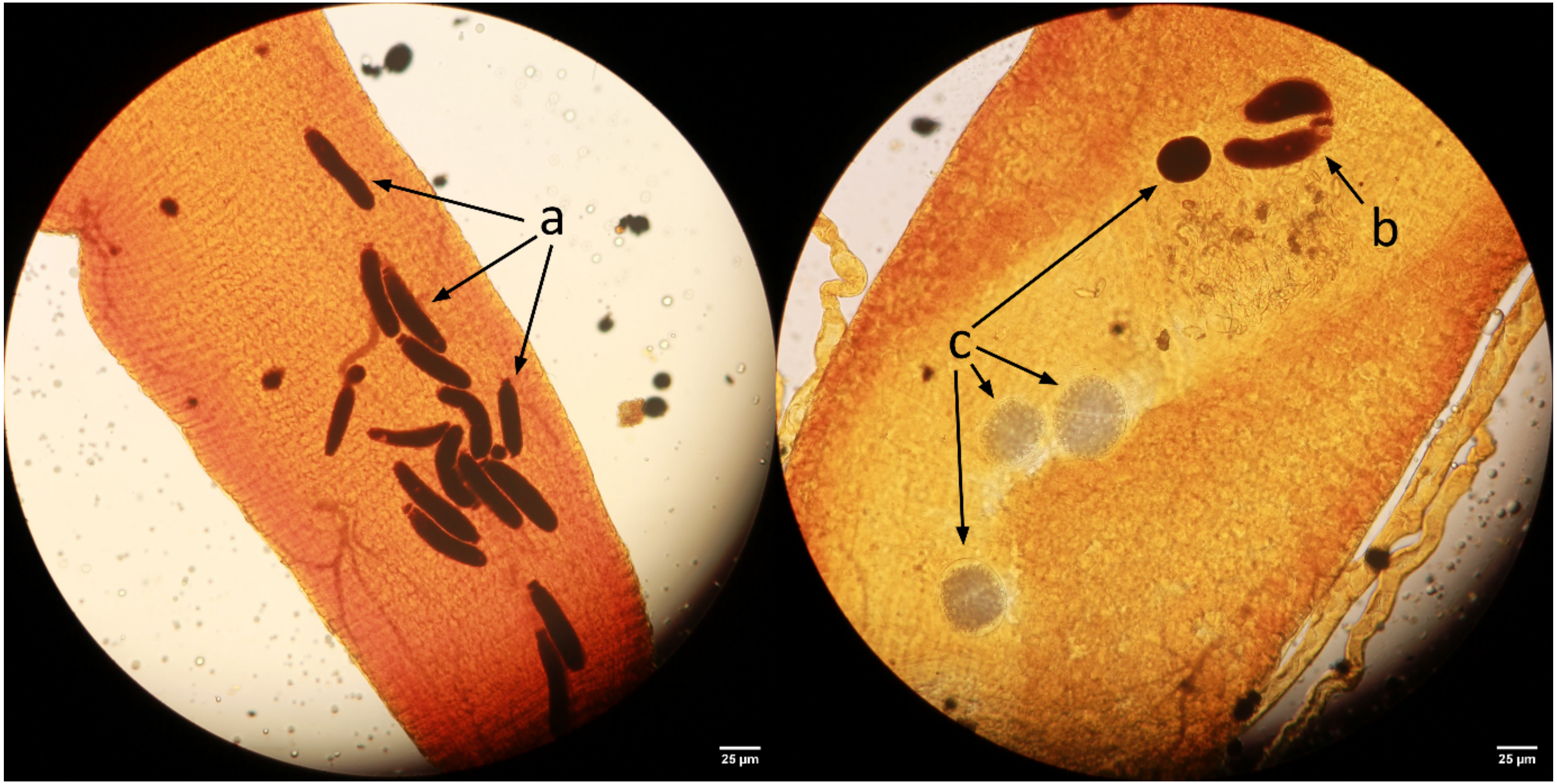

Figure S6. Microscope images of dissected larval *T. castaneum* guts containing trophonts (a), gamonts (b) and gametocysts (c) of the eugregarine parasite.

Supplementary Figure 7

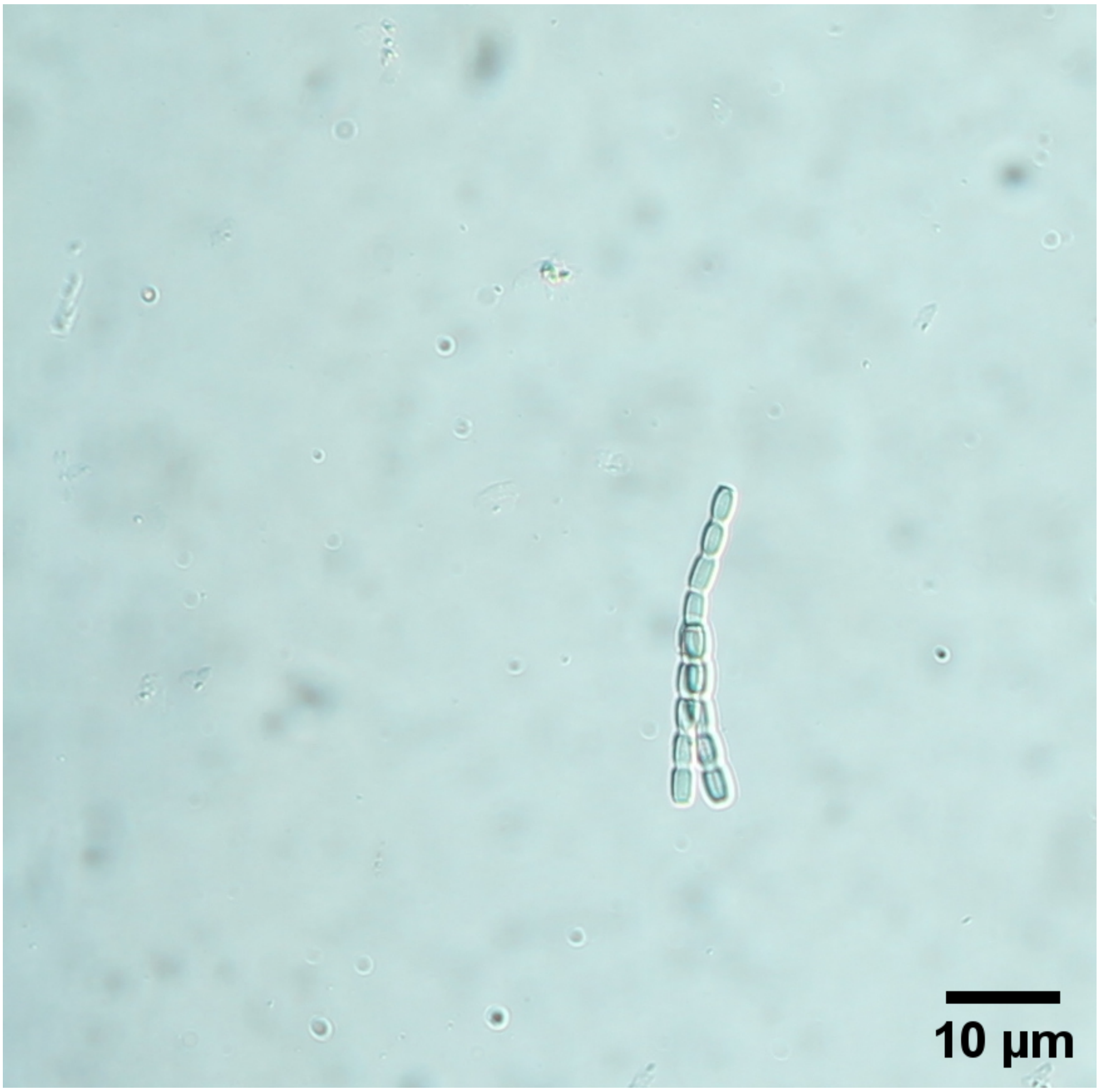

Figure S7. A chain of eugregarine oocysts.

Supplementary Figure 8

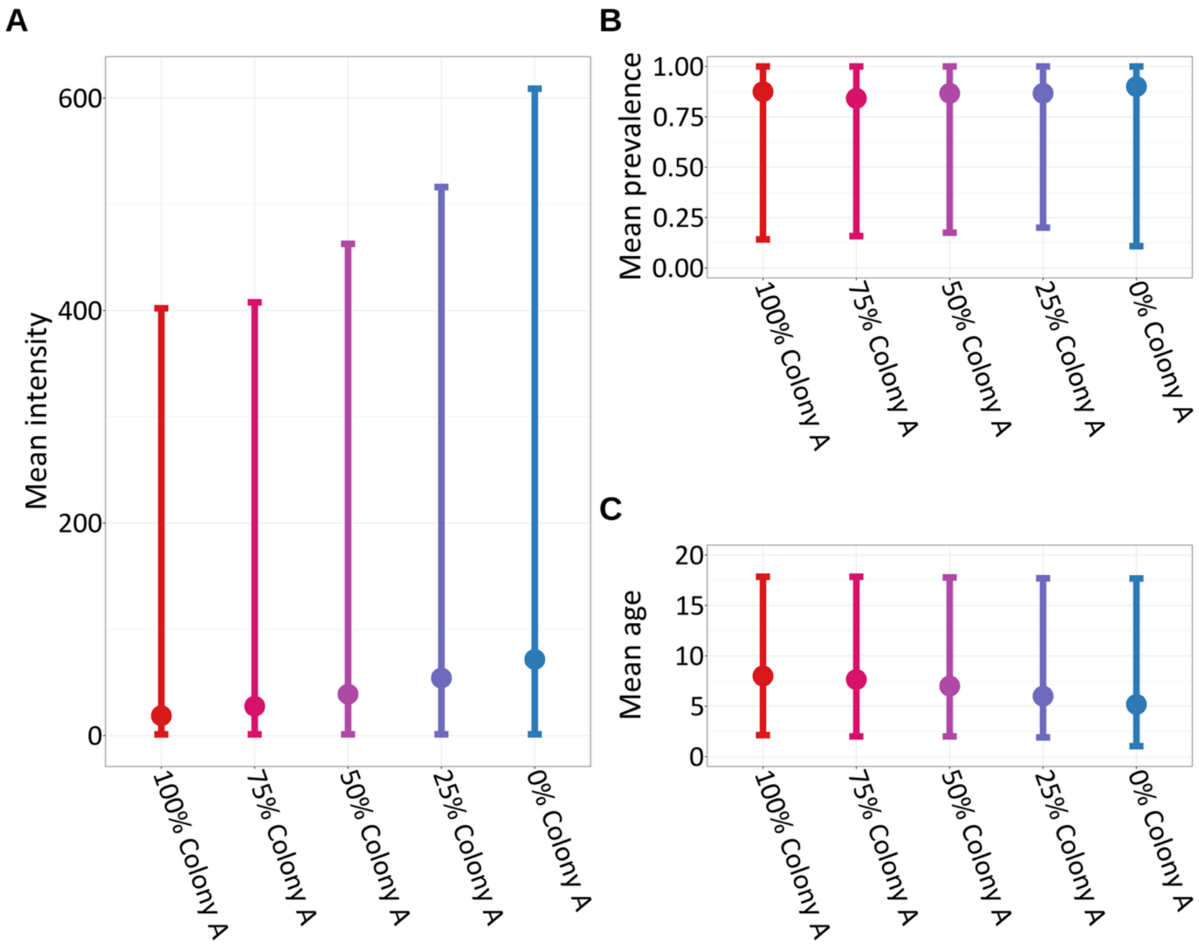

Figure S8. ABM initial simulation dynamics. Points show median (50th centile) values across the first 60 timesteps of 250 simulations for each treatment of population mean (A) intensity, (B) prevalence, and (C) agent age, for the different experimental scenarios. Error bars are 90% CIs (5th to 95th centiles) of the predicted mean equilibrium values (timesteps 0-60) across the 250 simulations.

Supplementary Figure 9

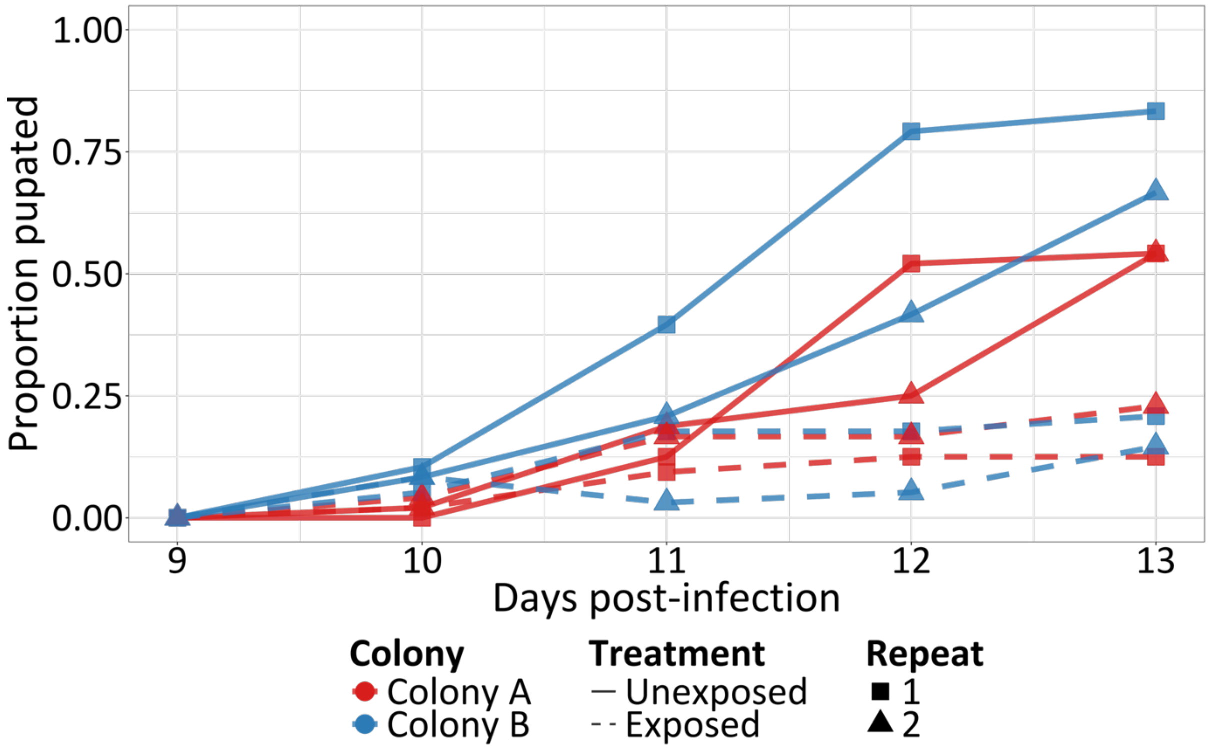

Figure S9. Proportion of larvae pupated over time (a proxy for development rate) for each colony. Unexposed larvae developed significantly faster than exposed larvae. Colony is denoted by colour, experimental repeat is denoted by point shape, treatment is denoted by line type.

Supplementary Figure 10

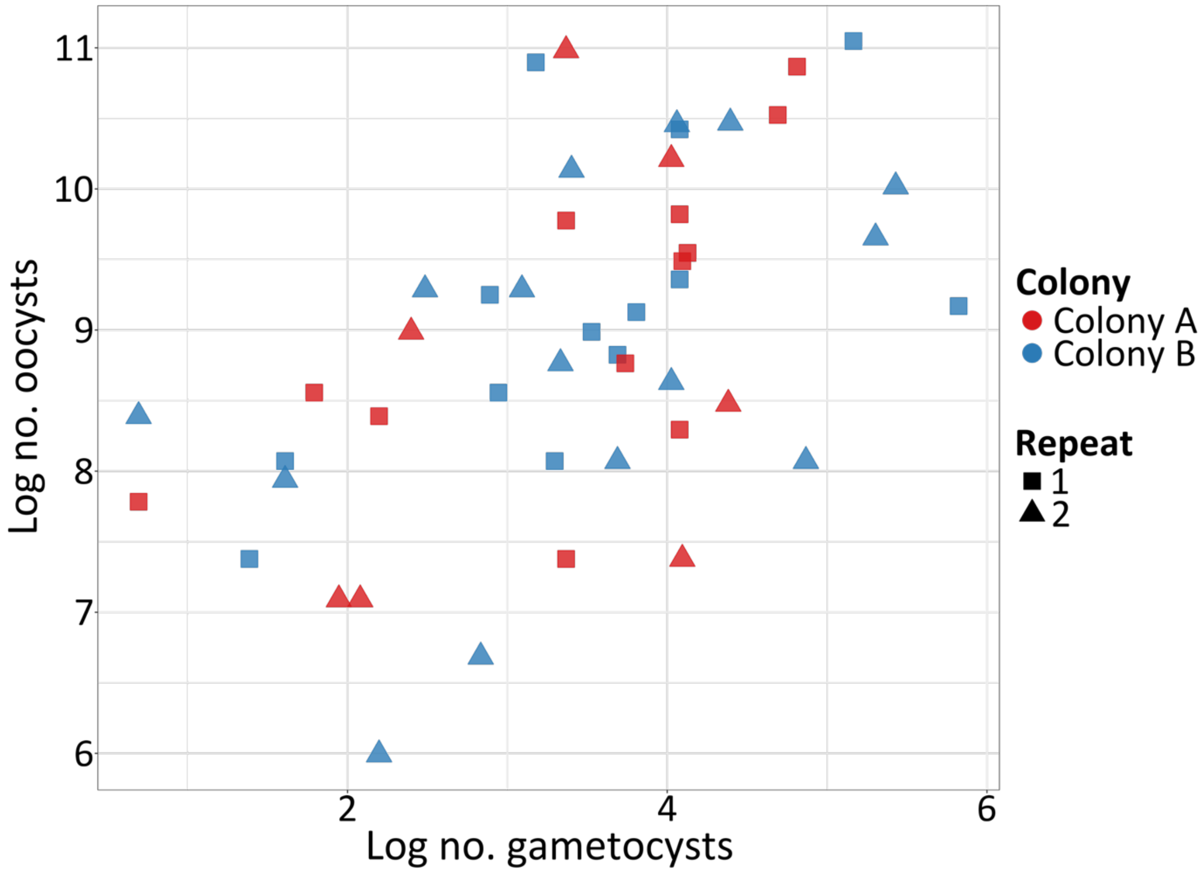

Figure S10. Log number of gametocysts vs. log number of oocysts per individual for each colony. There was a positive relationship between the two measures. Colony is denoted by colour and experimental repeat is denoted by point shape.

Supplementary Figure 11

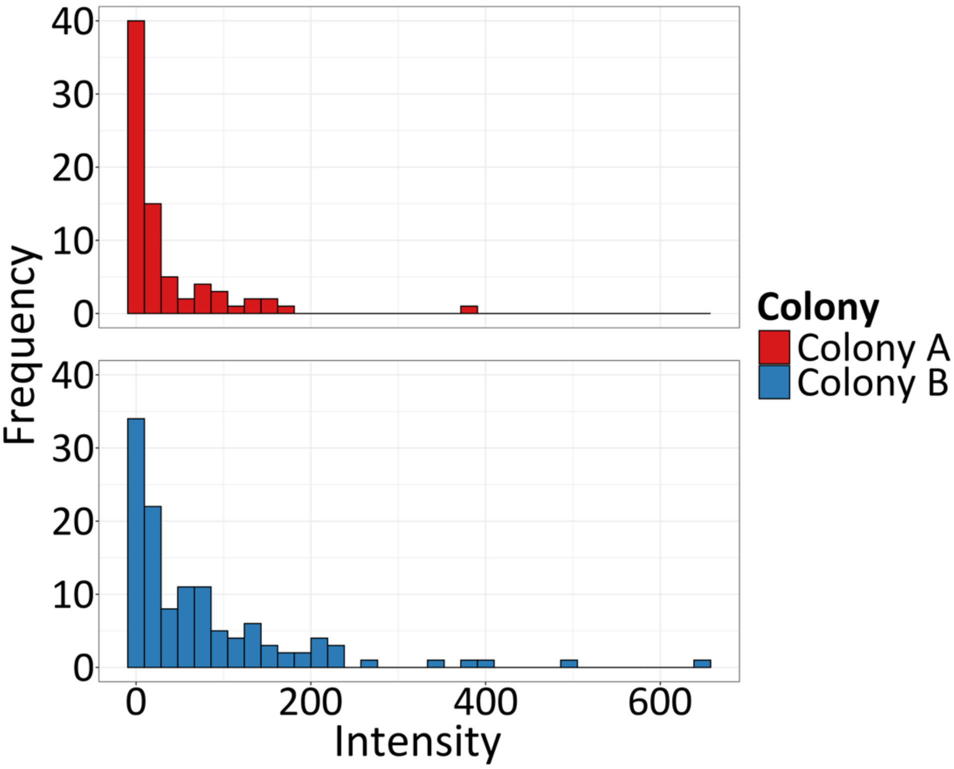

Figure S11. Frequency histograms for infection intensity for each colony. Colony A had a significantly lower variance than Colony B. Colony is denoted by colour. Bin width is 20 units.

Supplementary Figure 12

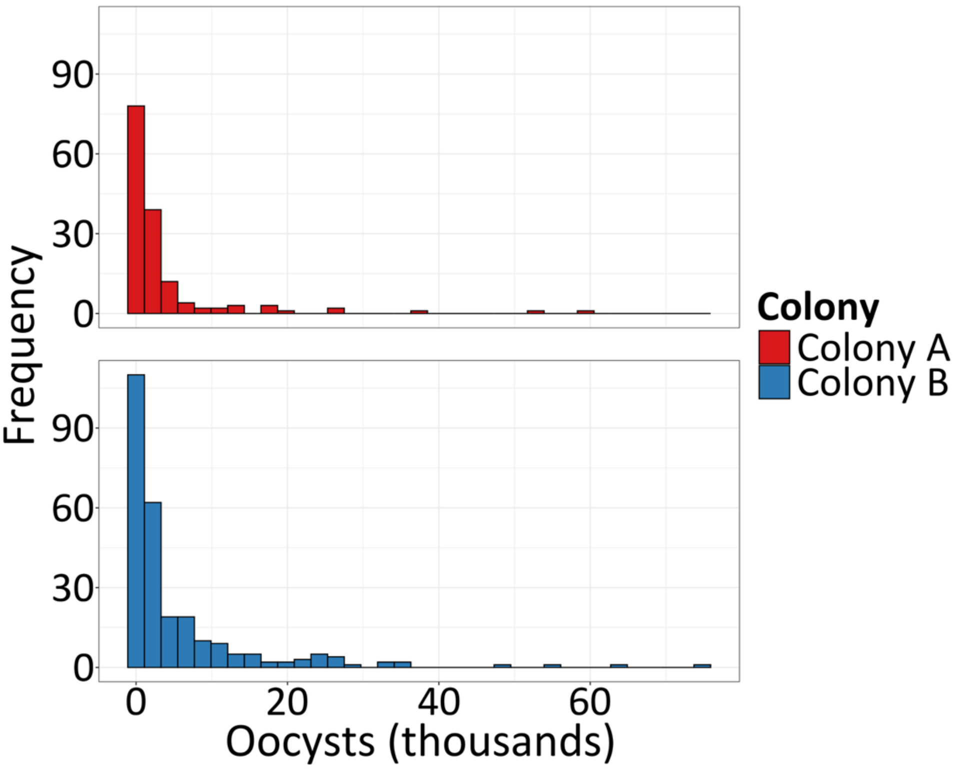

Figure S12. Frequency histograms for infectiousness (in thousands of oocysts) for each colony. Colony A had a significantly lower variance than Colony B. Colony is denoted by colour. Bin width is 2000 units.

Supplementary Table 1

Table S1. Pairwise comparisons of differences between repeats for oocyst shedding. Each cell reports the estimate (top value) and the p-value (bottom value). When the estimate is positive, the experimental repeat in the column has the higher oocyst count than the experimental repeat in the row. When the estimate is negative, the experimental repeat in the column has the lower oocyst count than the experimental repeat in the row. Cells highlighted in grey are statistically significant.

	**Repeat 1**				
**Repeat 2**	0.67442<br>0.4532	**Repeat 2**			
**Repeat 3**	1.96611<br><0.0001	1.29169<br>0.0012	**Repeat 3**		
**Repeat 4**	2.01419<br><0.0001	1.33977<br>0.0010	0.04808<br>0.9998	**Repeat 4**	
**Repeat 5**	1.97155<br><0.0001	1.29713<br>0.0011	0.00543<br>1.0000	-0.04264<br>0.9999	**Repeat 5**
**Repeat 6**	1.75001<br>0.0026	1.07559<br>0.2301	-0.21610<br>0.9958	-0.26418<br>0.9900	-0.22153<br>0.9953

Supplementary Table 2

Table S2. Results of *post hoc* EMMs test for differences in mean prevalence between experimental treatments. Each cell reports the estimate (top value) and the p-value (bottom value). When the estimate is positive, the treatment in the column has the higher mean prevalence than the treatment in the row. When the estimate is negative, the treatment in the column has the lower mean prevalence than the treatment in the row. Cells highlighted in grey are statistically significant.

	**100% Colony A**			
**75% Colony A**	-0.32843<br>0.2155	**75% Colony A**		
**50% Colony A**	-0.33099<br>0.2099	-0.00257<br>1.0000	**50% Colony A**	
**25% Colony A**	-0.43185<br>0.0478	-0.10343<br>0.9672	-0.10086<br>0.9702	**25% Colony A**
**0% Colony A**	-0.51280<br>0.0117	-0.18438<br>0.7865	-0.18181<br>0.7960	-0.08095<br>0.9879

Supplementary Table 3

Table S3. Results of *post hoc* EMMs test for differences in mean intensity between experimental treatments. Each cell reports the estimate (top value) and the p-value (bottom value). When the estimate is positive, the treatment in the column has the higher mean intensity than the treatment in the row. When the estimate is negative, the treatment in the column has the lower mean intensity than the treatment in the row. Cells highlighted in grey are statistically significant.

	**100% Colony A**	**75% Colony A**	**50% Colony A**	**25% Colony A**
**75% Colony A**	-0.299595<br>0.0174			
**50% Colony A**	-0.300083<br>0.0177	-0.000488<br>1.0000		
**25% Colony A**	-0.564438<br><0.0001	-0.264843<br>0.0369	-0.264355<br>0.0365	
**0% Colony A**	-0.478002<br><0.0001	-0.178427<br>0.3149	-0.177939<br>0.3206	0.086416<br>0.8852

Supplementary Table 4

Table S4. Amount of flour and generation of adults (since establishment in Liverpool) used to lay eggs in each experimental repeat of the susceptibility assay experiment.

Experimental repeat no.	Amount of parasite-contaminated flour	Adult generation
1	7.8g	3
2	6.5g	4
3	7.0g	5

Supplementary Table 5

Table S5. Details for differences between experimental repeats for the infectiousness assay experiment.

Experimental repeat no.	Adult generation	Number of larvae per colony	Parasite-contaminated flour (g)	Negative control	Parasite-free flour (g)	Days post-exposure that larvae were removed and placed in individual wells of a 96-well plate	Number of larvae removed per day
1	5	250	8	130 larvae on parasite-free flour	10	8, 9, 10, 11	48
2	5	250	8	130 larvae on parasite-free flour	10	8, 9, 10, 11	48
3	8	1,000	15	20 larvae from each colony dissected on Day 0 to confirm that all larvae were parasite free	15	9, 10, 11	96
4	8	1,000	15	20 larvae from each colony dissected on Day 0 to confirm that all larvae were parasite free	15	9, 10, 11	96
5	8	1,000	15	20 larvae from each colony dissected on Day 0 to confirm that all larvae were parasite free	15	9, 10, 11	96
6	8	1,000	15	20 larvae from each colony dissected on Day 0 to confirm that all larvae were parasite free	15	4, 5, 6, 7	96

Supplementary Table 6

Table S6. Details of parameters for the ABM.

Parameter	Description	Value
P_0	Initial number of parasite infective stages in the environment	4000
$P_{exp.}$	Parasite exposure rate (per agent, per timestep)	0.00001
$P_{mort.}$	Parasite infective stage mortality rate (per timestep)	0.10
$t_{pup.}$	Expected mean time to pupation (timesteps)	23
σ_A	Mean susceptibility value for Colony A, defined as the mean probability of parasite establishment (per parasite, per agent, per timestep)	0.26
σ_B	Mean susceptibility value for Colony B, defined as the mean probability of parasite establishment (per parasite, per agent, per timestep)	0.42
ι	Mean infectiousness value for all agents	30
$Day\ length$	Number of model ticks per day	1
K	Agent carrying capacity	120
$No.Colony\ A$	The number of larvae in the simulation from Colony A. The number of Colony B larvae was calculated as ($K - No.Colony\ A$)	Variable

Supplementary Table 7

Table S7. Within-colony means, variances, variance:mean ratios and coefficients of variation (standard deviation/mean) for intensity.

Experimental Repeat	Colony	Mean	Variance	Variance:mean ratio	Coefficient of variation
1	A	60.04	7867.9	131.04	1.48
	B	67.86	12606.8	185.78	1.65
2	A	7.83	57.3	7.31	0.97
	B	79.79	8959.0	112.28	1.19
3	A	32.52	1843.8	56.70	1.32
	B	82.40	11190.1	135.81	1.28

Supplementary Table 8

Table S8. Within-colony means, variances, variance:mean ratios and coefficients of variation (standard deviation/mean) for infectiousness.

Experimental Repeat	Colony	Mean	Variance	Variance:mean ratio	Coefficient of variation
1	A	14733	241362424	16382	1.05
	B	16862	406049231	24081	1.20
2	A	14686	463344762	31551	1.47
	B	12107	141839238	11716	0.98
3	A	1764	6432135	3647	1.44
	B	3684	28565029	7755	1.45
4	A	1571	11459894	7293	2.15
	B	6497	175344900	26990	2.04
5	A	2183	14723227	6745	1.76
	B	3446	26145375	7587	1.48
6	A	3009	34194466	11365	1.94
	B	6242	74549394	11942	1.38

References

1. J. D. Logan, J. Janovy Jr, B. E. Bunker, The life cycle and fitness domain of gregarine (Apicomplexa) parasites. *Ecological Modelling* **233**, 31-40 (2012).
2. R. E. Clopton, "Order Eugregarinorida Léger 1900" in The Illustrated Guide to the Protozoa, J. J. Lee, G. F. Leedale, P. Bradbury, Eds. (Society of Protozoologists, Lawrence, Kansas, USA, 2000), vol. 1, pp. 205-288.
3. A. M. Thomas, V. H. Rudolf, Challenges of metamorphosis in invertebrate hosts: maintaining parasite resistance across life-history stages. *Ecological Entomology* **35**, 200-205 (2010).
4. J. T. Critchlow, A. Norris, A. T. Tate, The legacy of larval infection on immunological dynamics over metamorphosis. *Philosophical Transactions of the Royal Society B: Biological Sciences* **374**, 20190066 (2019).
5. A. Criado-Fornelio *et al.*, A survey for gregarines (Protozoa: Apicomplexa) in arthropods in Spain. *Parasitology Research* **116**, 99-110 (2017).
6. S. Rueckert, D. Devetak, Gregarines (Apicomplexa, Gregarinasina) in psocids (Insecta, Psocoptera) including a new species description and their potential use as pest control agents. *European Journal of Protistology* **60**, 60-67 (2017).
7. J. Janovy, J *et al.*, New and emended descriptions of gregarines from flour beetles (Tribolium spp. and Palorus subdepressus: Coleoptera, Tenebrionidae). *Journal of Parasitology* **93**, 1155-1170 (2007).
8. R. E. Clopton, Phylogenetic relationships, evolution, and systematic revision of the septate gregarines (Apicomplexa: Eugregarinorida: Septatorina). *Comparative Parasitology* **76**, 167-190 (2009).
9. E. Vivier, I. Desportes, "Phylum Apicomplexa" in Handbook of Protoctista, L. Margulis, J. O. Corliss, M. Melkonian, D. J. Chapman, Eds. (Jones and Bartlett Publishers, Boston, MA, 1990), chap. 30, pp. 549-573.
10. P. Leslie, T. Park, The intrinsic rate of natural increase of Tribolium castaneum Herbst. *Ecology* 10.2307/1932450, 469-477 (1949).
11. R Core Team (2022) R: A language and environment for statistical computing. (R Foundation for Statistical Computing, Vienna, Austria).
12. D. Bates, M. Mächler, B. Bolker, S. Walker, Fitting Linear Mixed-Effects Models Using lme4. *Journal of Statistical Software* **67**, 1-48 (2015).
13. W. Venables, B. Ripley (2002) Modern Applied Statistics with S. (Springer, New York).
14. M. McGillycuddy, D. Warton, G. Popovic, B. Bolker, Parsimoniously fitting large multivariate random effects in glmmTMB. *Journal of Statistical Software* 10.18637/jss.v112.i01 (2025).
15. F. Hartig (2022) DHARMa: Residual Diagnostics for Hierarchical (Multi-Level / Mixed) Regression Models.
16. R. V. Length (2023) emmeans: Estimated Marginal Means, aka Least-Squares Means.
17. H. Wickham, Reshaping data with the reshape package. *Journal of Statistical Software* **21** (2007).
18. J. Fox, S. Weisberg (2019) An R Companion to Applied Regression. (Sage, Thousand Oaks, CA).
19. H. Wickham *et al.*, Welcome to the tidyverse. *Journal of Open Source Software* 10.21105/joss.01686 (2019).
20. U. Wilensky (1999) NetLogo. (Center for Connected Learning and Computer-Based Modeling, Northwestern University, Evanston, IL).
21. H. Wickham (2016) ggplot2: Elegant Graphics for Data Analysis. (Springer-Verlag New York).
22. Y. Qiu (2022) showtext: Using Fonts More Easily in R Graphs.
23. A. Kassambara (2023) ggpubr: 'ggplot2' Based Publication Ready Plots.
24. E. Clarke, S. Sherrill-Mix, C. Dawson (2023) ggbeeswarm: Categorical Scatter (Violin Point) Plots.

25. C. Ahlmann-Eltze, I. Patil, ggsignif: R Package for Displaying Significance Brackets for 'ggplot2'. *PsyArxiv* doi:10.31234/osf.io/7awm6 (2021).
26. T. L. Pedersen (2022) patchwork: The Composer of Plots.
